# What characterizes the exceptional cognition of superagers? A systematic review of multidomain biomarkers of successful cognitive aging

**DOI:** 10.1093/geront/gnaf277

**Published:** 2025-11-24

**Authors:** Yiru Yang, Xiaolei Li, Shudan Gao, Yuanxu Gao

**Affiliations:** School of Nursing and Rehabilitation, Cheeloo College of Medicine, Shandong University, Jinan, Shandong, China; School of Nursing and Rehabilitation, Cheeloo College of Medicine, Shandong University, Jinan, Shandong, China; Shandong Provincial Key Laboratory of Brain Science and Mental Health, Faculty of Psychology, Shandong Normal University, Jinan, China; Institute for AI in Medicine and Faculty of Medicine, Macau University of Science and Technology, Macau, China

**Keywords:** Cognitive aging heterogeneity, Resilience, Successful aging, Neuroimaging, Brain reserve

## Abstract

**Background and Objectives:**

Substantial heterogeneity in cognitive aging trajectories has been observed among older adults, with some individuals maintaining exceptional cognitive function (“superagers” or “successful cognitive aging [SCA]”). The biological mechanisms underlying SCA remain unclear. This systematic review synthesizes current evidence on quantifiable SCA biomarkers to address this critical gap.

**Research Design and Methods:**

Following the Preferred Reporting Items for Systematic Reviews and Meta-Analyses guidelines, we systematically searched PubMed, Scopus, PsycINFO, and Web of Science (up to December 2024). After screening 6,699 records, 62 studies met the inclusion criteria. Data from included studies were extracted, assessed for risk of bias, and synthesized for integrated findings.

**Results:**

We identified 34 SCA definitions, categorized them into three types, and analyzed biomarkers across six domains: (1) genetic/epigenetic biomarkers, (2) biofluid biomarkers, (3) histological biomarkers, (4) positron emission tomography biomarkers, (5) structural magnetic resonance imaging biomarkers, and (6) functional neuroimaging biomarkers. Integrated findings suggest that SCA is driven by unique multidomain biological mechanisms (e.g., young DNA methylation age, high von Economo neuron density, and efficient glucose metabolism, etc.), not merely resistance to age-related neuropathology such as amyloid-β and tau. Neuroimaging findings highlight the role of brain reserve, maintenance, and compensation on SCA, particularly within a newly defined “cingulate gyrus–medial temporal lobe–frontal cortex” brain signature.

**Discussion and Implications:**

This systematic review advances our understanding of SCA’s biological substrates, provides theoretical frameworks for future SCA biomarker research, and offers a foundation for future strategies to promote cognitive health in aging populations.

The aging population is rapidly increasing worldwide, posing significant challenges to public health and social services (Dogra et al., 2022), particularly due to presently incurable age-related cognitive impairments like Alzheimer’s disease (AD) (Scheltens et al., 2021). While cognitive decline resulting in cognitive impairment was once considered inevitable, research now highlights substantial heterogeneity in cognitive aging trajectories (Lindenberger, 2014; Nyberg et al., 2020). Notably, some older adults maintain exceptional cognitive function, termed “superagers” or “successful cognitive aging (SCA)” (Harrison et al., 2012; Krivanek et al., 2021). Beyond preventing cognitive disease, achieving graceful aging and high-quality longevity is a social priority, driving scientific interest in SCA’s underlying mechanisms (Nyberg & Pudas, 2019).

Biomarker research has provided comprehensive insights into the biological underpinnings of cognitive aging, especially pathological cognitive aging, for cognitive disease diagnosis and treatment (Jack et al., 2024; Scheltens et al., 2021). Despite genetic, histological, and biochemical analyses of biofluids or in vitro tissue samples, advancements in neuroimaging techniques, including magnetic resonance imaging (MRI), electroencephalography, event-related potential (ERP), and positron emission tomography (PET), etc., have particularly revolutionized our understanding of brain aging and serve as powerful tools for discovering biomarkers. Various approaches have been employed to explore the distinct biological characteristics of SCA, aiming at identifying reliable biomarkers (de Godoy et al., 2021a). These biomarkers could not only potentially serve as early indicators of the cognitive trajectory for targeted cognitive enhancement (Lindenberger, 2014) but also inform new strategies against age-related cognitive impairment (Mapstone et al., 2017; Santangelo et al., 2022).

Despite the growing attention in this field, several crucial issues remain unsolved. First, a consensus definition of SCA is not available, resulting in difficulty in taking full advantage of biomarkers for group identification. Much more work is needed to establish the biological criteria of SCA individuals, similar to the AT(N) system of AD (Jack et al., 2024). A systematic review of existing definitions and biomarkers is a critical first step. Second, it is unknown whether SCA reflects resistance to pathology or unique preservation mechanisms. Some studies associate SCA with lower tau pathology and reduced risk of conversion to cognitive impairment (Dang et al., 2019a; Gefen et al., 2021), while others highlight distinct neural signatures of successful and pathological cognitive aging (Arenaza-Urquijo et al., 2019; Yang et al., 2022). Third, most existing SCA reviews focus on definitions and behavioral influencing factors, including demographical factors, physical conditions, mental health, lifestyles, and so on (Krivanek et al., 2021; Nyberg et al., 2020); with few examining biomarkers (Borelli et al., 2018; de Godoy et al., 2021a), and existing biomarker reviews are narrow in scope (*n* = 9 and 21, separately) and lack synthesis across domains. Finally, the heterogeneous nature of cognitive aging is demonstrated (Loaiza, 2024), and a “successful–usual–pathological cognitive aging” conceptual framework has been proposed (Nyberg & Pudas, 2019), where the mechanism explorations of successful and pathological cognitive aging are theoretically equally important for ameliorating age-related cognitive deficits. Currently, however, the relatively few explorations and summaries of the underlying mechanisms of SCA skew research of cognitive aging heterogeneity toward pathological cognitive aging, which, to some extent, exacerbates older adults’ fear of ageing and raises the issue of possible ageism, which is detrimental to the promotion of healthy aging (Nelson, 2016).

A comprehensive integration of multidomain biomarkers is critically needed to elucidate the underlying mechanisms of SCA, particularly given the current paucity of systematic reviews in this field. This systematic review synthesizes current evidence on SCA biomarkers, including genetic, histological, biochemical, and neuroimaging, aiming to answer the aforementioned questions. By clarifying the biological mechanisms of SCA, we aim to inspire targeted strategies for preventing cognitive impairment and promoting cognitive health.

## Method

This systematic review followed the standard guidelines of Preferred Reporting Items for Systematic Reviews and Meta-Analyses (PRISMA) 2020 (Page et al., 2021) and was registered with PROSPERO (International Prospective Register of Systematic Reviews, ID: CRD42024578134).

### Literature search strategy

Four databases (PubMed, Scopus, PsycINFO, and Web of Science) were searched from inception to December 15, 2024, and the search terms were as follows: (“successful cognitive aging” OR “exceptional aging” OR “superaging” OR “super-aging” OR “superager” OR “supernormal” OR “high performing” OR “cognitive resilience” OR “exceptional cognition” OR “superior cognition” OR “maintained cognition” OR “exceptional memory” OR “superior memory” OR “maintained memory”) AND (“biomarker” OR “neuroimaging” OR “magnetic resonance imaging” OR “positron emission tomography” OR “biology” OR “pathology” OR “histology” OR “gene” OR “metabolism”). Two independent researchers (Y.R.Y., X.L.L.) conducted the search, screened references, and resolved discrepancies by consensus.

### Inclusion and exclusion criteria

We focused on studies that explored potential biomarkers of SCA using genetic, histological, biochemical, neuroimaging, or other quantitative methods. The inclusion criteria for this study were as follows: (1) peer-reviewed, English-language articles; (2) participants aged 60 years and older; (3) SCA defined by neuropsychological tests; (4) at least one biomarker reported; and (5) original data analyzed. The exclusion criteria were as follows: (1) reviews and meta-analyses; (2) inappropriate records, including case studies, comments, editorials, conference abstracts, and preprints; and (3) inappropriate subject populations, including animals, young or middle-aged adults only, and specific patient groups.

### Study selection and data extraction

Records were screened by Y.R.Y. and X.L.L. independently, and discrepancies were solved by consensus. The search results were merged, and duplicates were deleted first. Titles and abstracts of remaining articles were read, and articles unrelated to the current theme and inappropriate ones were excluded according to the exclusion criteria. The included articles were identified after the aforementioned selection and imported into EndNote 21, and the references of them were all screened to identify additional records.

Data were extracted from each included study, including study details (title, authors, year of publication, journal, country, database), participant demographics (sample size, age, sex), neuropsychological tests and partition criteria used in SCA definitions, biomarker types (genetic/epigenetic, biofluid, histological, PET, structural MRI, and functional neuroimaging), and key findings, especially the statistically significant differences between the SCA group and the normal control group.

### Risk of bias and study quality assessments

The risk of bias and study quality were evaluated using the Quality Assessment of Diagnostic Accuracy Studies-2 (QUADAS-2) (Whiting et al., 2011) instrument. QUADAS-2 comprises four domains: patient selection, index test, reference standard, and flow and timing. All the domains are designed to assess the risk of bias, and the first three domains are also designed to evaluate concerns regarding applicability. Two researchers, Y.R.Y. and S.D.G., assessed the included papers, and discrepancies were solved by consensus. Each domain was considered to have either a “low” or “high” risk of bias, and “unclear” was used when insufficient information was available to permit a judgment.

### Data synthesis and visualization

First, we organized all SCA definitions, including neuropsychological tests and partition criteria, and synthesized these definitions according to study design (e.g., cross-sectional or longitudinal) and control group selection (e.g., same-age peers or younger adults) to identify typical types of definitions. Second, key findings of included studies were synthesized by six biomarker domains and labelled by definition types qualitatively for further comparison and induction. Third, for structural neuroimaging findings, we further identified all brain regions of interest (ROIs) showing significant preservation in SCA individuals in the included studies, and (1) gray matter (GM) ROIs were mapped to the Harvard-Oxford atlas (for cortical regions) and the Automatic subcortical segmentation subcortical atlas (for subcortical regions) using the “ggseg” package (https://ggseg.github.io/ggseg/) of R software (version 4.2.0), the data and code can be found at https://github.com/ariayryang/SCA-signature; (2) white matter (WM) ROIs were visualized based on the JHU WM tractography atlas via BrainNet Viewer software (https://www.nitrc.org/projects/bnv/).

## Results

### General characteristics and quality assessment

**Figure 1.**
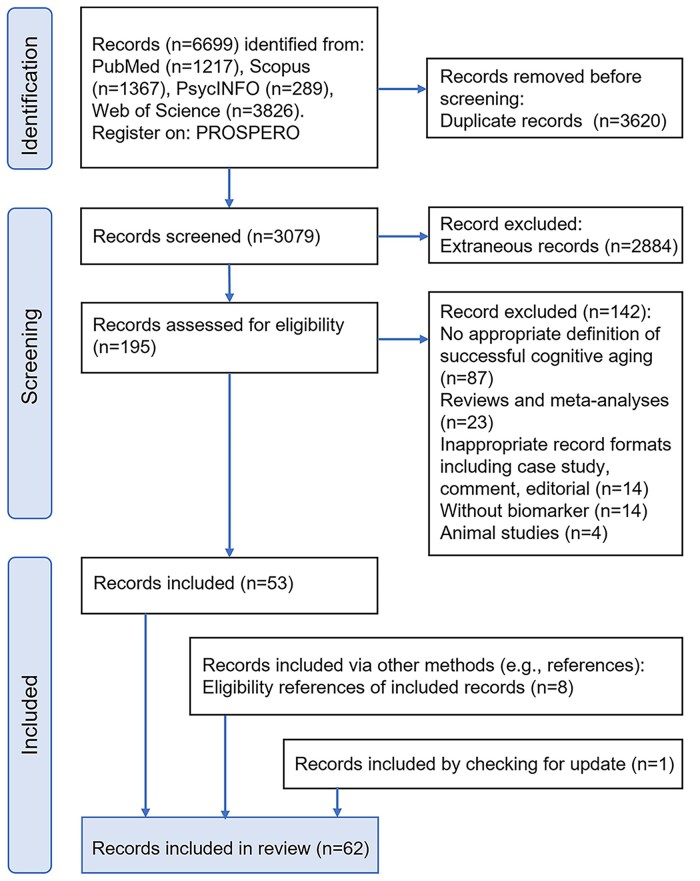
Flow chart of study screening and selection under the guidance of the PRISMA 2020.

The PRISMA-guided selection process (Figure 1) identified 6,699 records from four databases. After removing 3,620 duplicates and 2,884 irrelevant records, 195 full-text articles were screened, with 142 excluded according to the criteria. Three researchers (Y.R.Y., X.L.L., S.D.G.) independently evaluated 53 eligible articles, supplemented by eight additional records from reference screening and one added by checking for updates, yielding 62 included studies (Table S1). The ­general characteristics of the included studies are summarized in Table S2. Most studies (*n* = 51) were published after 2015, with 32 published after 2020, reflecting growing interest in the biological mechanism of SCA. The sample sizes of SCA participants varied widely (range: 5–1,060; median = 34; mean ± *SD* = 94.64 ± 164.68). We identified 14 distinct terms describing SCA populations, with “superager” being the most common (*n* = 27). Geographically, studies primarily came from the United States (*n* = 36), followed by South Korea (*n* = 5), China (*n* = 4), and several other countries. Forty-seven studies utilized established aging cohorts, notably the Alzheimer’s Disease Neuroimaging Initiative (*n* = 14) and the Northwestern SuperAging Program (*n* = 8).

**Figure 2.**
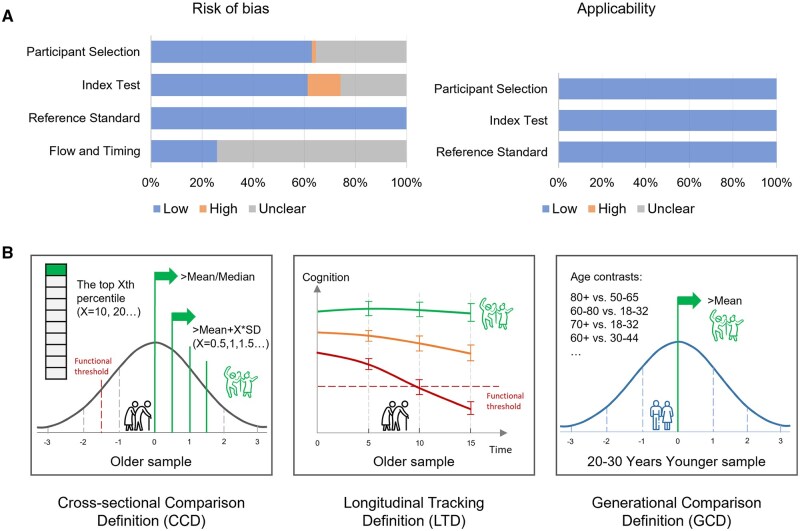
(A) Quality assessment results of included studies; the data are consistent with Table S3. (B) The “three-type” framework of successful cognitive aging definitions.

QUADAS-2 assessment revealed a generally low risk of bias (Figure 2A, Table S3), with several exceptions. One study showed high patient selection bias from convenience sampling; eight studies had high index test bias due to unblinded biomarker assessment with knowledge of the results of grouping, and 16 studies provided insufficient information for clear judgments. Most studies (*n* = 46) had an unclear risk of bias in flow and timing because the information was incomplete to estimate if the interval between the index test and reference standard was inappropriate. By strictly enforcing the inclusion and exclusion criteria, the risks of applicability of included studies were all low and acceptable.

### Definitions of SCA

We identified 34 distinct SCA definitions across 62 studies (Table 1), and the inconsistency of SCA definition is self-evident. We divided these definitions into three types (Figure 2B): (1) cross-sectional comparison definition (CCD, 14 definitions in 19 studies), identifying top cognitive performers compared with their peers; (2) longitudinal tracking definition (LTD, 8 definitions in 14 studies), identifying high cognitive maintainers longitudinally; and (3) generational comparison definition (GCD, 11 definitions in 28 studies), identifying older adults who had equivalent cognition to adults 20–30 years younger. Additionally, another study identified SCA using more than one method. Memory (54 studies) and executive function (39 studies) were most frequently assessed when defining SCA, typically using the Rey Auditory Verbal Learning Test (AVLT, 25 studies) and the Trail-Making Test Part B (TMT-B, 31 ­studies) (Table S4).

**Table 1.** Definitions of successful cognitive aging used in the included studies.

Publications	Thresholds	Definitions
***Cross-sectional comparison definition***		
**Fjell et al. (**2006)	Median score	**High fluid function and high executive function old group**: dividing the sample at the median score.
**Daffner et al. (**2006)**, ** **Riis et al. (**2008)	The 67th percentile	**Cognitively high performer**: scored above the 67th percentile according to published age-matched norms on four of six cognitive tests.
**Waiter et al. (**2008)	Mean + 0.5 *SD*	**Cognitive sustainer**: childhood IQ 85–115, scored at least 0.5 *SD* above the mean on RPM at age about 68.
**Silverman et al. (**2012)	The 10th percentile	**Successful cognitive aging**: aged 75+ or 85+ (two cohorts), CDR = 0, MMSE score better than the 10th percentile of age- and education-adjusted norms.
**Barral et al. (**2014)**, ** **Patel et al. (**2024)	Mean + 1.5 *SD*	**Exceptional EM**: 1.5 *SD* above the demographically adjusted mean EM score in the offspring generation.
**Mapstone et al. (**2017)	Mean ± 1.35 *SD*	**Supernormal**: age > 70, memory *Z*-score >1.35 (the 90th percentile), and all other domain composite *Z*-scores > −1.35 or greater than the 10th percentile.
**Dekhtyar et al. (**2017 **)**	The 20th percentile	**Optimal Memory Performer**: age ≥ 75, top 20% (≥ mean + 0.5 *SD*) for memory composite score.
**Park et al. (**2021, 2022b **)**	Mean	**Successful cognitive aging**: scores for global cognition, memory, attention, and executive function were all above the average of normative values.
**Dominguez et al. (**2024)**, ** **Dominguez et al. (**2021)	The 50th percentile	**Top cognitive performance**: age ≥ 60/70/90 (three cohorts), performance in the top 50th percentile of memory and executive function.
**Jia et al. (**2022 **)**	Mean + 1 *SD*, Mean ± 1 *SD*	**Superager:** performing> 1 *SD* of the mean score on memory, and performing within 1 *SD* of the average range on other domains.
**de Souza et al. (**2022 **)**	Mean + 1.5 *SD*	**Superager:** age ≥ 80, with memory score > 1.5 *SD* and standard scores for nonmemory domains.
**Linuma et al. (**2022)**, ** **Tobe et al. (**2023 **)**	Median score	**High cognitive function group**: higher than the median of their total Five-Cog score.
**Yang et al. (**2022 **)**	Mean + 1.5 *SD*, Mean − 1.5 *SD*	**Successful cognitive aging**: age > 70, scored higher than mean + 1.5 *SD* on memory or executive function, and ≥mean − 1.5 *SD* in all other tests.
**Biswas et al. (**2023 **)**	MMSE > 28	**Superior global cognitive performer**: MMSE score ≥ 28 in the last visit 12 to 2 months before death.
***Longitudinal tracking definition***		
**Rosano et al. (**2012 **)**	Slope > 0; 9 years	**Maintainers:** 3MS score slope > 0 calculated by the repeated 3MS measures in about 9 years.
**Degerman et al. (**2017)**, ** **Josefsson et al. (**2012)**, ** **Pudas et al. (**2013)	LC; 15 years	**Maintainers**: a moderate to high baseline memory score and a better-than-average rate of change in 15 years.
**Lin et al. (**2017a **)**	Mean + 1.5 *SD*, 2.56–3.33 years	**Supernormal**: having ADNI-MEM >1.5 across all the available clinical assessment visits with at least one follow-up assessment.
**Baran et al. (**2018)**, ** **Chen et al. (**2020)**, ** **Lin et al. (**2017b)**, ** **Wang and Zhang (**2021)**, ** **Wang et al. (**2019 **)**	LC; 5 years	**Supernormal**: mean age = 75 years old, keep high and stable EM and executive function composite scores over 5 years.
**Arenaza-Urquijo et al. (**2019)	Normal; 5 years	**Cognitively stable 80+**: 80+, maintained normal cognition for an average of 5 years (2–10 years).
**Chen et al. (**2022 **)**	LC, 4 years	**Successful ager**: LC models estimated both the baseline and longitudinal performance for four cognitive domains.
**Klinedinst et al. (**2023)	LC, 8 years	**Superager**: 12 latent groups were characterized, and 8 were carried forward, among which 2 groups were superagers (younger & older)
**Harrison et al. (**2024 **)**	Slope ≥ 0; 6.1 years	**Cognitive maintainer:** For all cognitive slopes, larger than or equal to 0.
***Generational comparison definition***		
**Borelli et al. (**2021)**, ** **de Godoy et al. (**2023, 2021b)**, ** **Diamond et al. (**2024)**, ** **Gefen et al. (**2021)**, ** **Gefen et al. (**2018)**, ** **Gefen et al. (**2015)**, ** **Harrison et al. (**2012)**, ** **Huentelman et al. (**2018)**, ** **Janeczek et al. (**2018)**, ** **Nassif et al. (**2022)**, ** **Spencer et al. (**2022 **)**	80+ *versus* 50–65	**Superager**: age >80, with EM performance at least as good as normative values for 50- to 65-year-olds, and were required to perform within or above 1 *SD* of the average range for their age for cognitive tests in other domains.
**Katsumi et al. (**2021)**, ** **Sun et al. (**2016)**, ** **Zhang et al. (**2020 **)**	60–80 *versus* 18–32	**Superager**: aged 60–80, performed ≥ the mean for adults aged 18–32 on EM and performed ≥ mean − 1 *SD* for their age group on executive function.
**Bott et al. (**2017)	60–80 *versus* 20–30	**Resilient-ager**: baseline processing speed was within 1.25 *SD* of the young adult comparison group and changed no more than 0.5 *SD* at follow-up.
**Harrison et al. (**2018)	70+ *versus* 18–32	**Successful ager**: age > 70, memory performance ≥ the mean of those aged 18–32 years, and normal-for-age performance on executive function.
**Dang et al. (**2019a, 2019b)	60+ *versus* 30–44	**Superager**: age >60, performed > the mean for 30–44-year-olds on memory and > mean − 1 *SD* using published normative data for all nonmemory tests.
**Kim et al. (**2020, 2024)**, ** **Park et al. (**2022a)	60+ *versus* 45-year-olds	**Superager:** memory ≥ average normative values of 45-year-olds on both memory tests, and at least average for age in other cognitive domains.
**Gardener et al. (**2021)	70+ *versus* 30-44	**Superior cognitive performance**: aged 70+, attained mean memory for individuals aged 30–44, and scored > mean − 1.5 *SD* for all other domains.
**Katsumi et al. (**2022)	70+ *versus* 16–29	**Superager**: age >70, performed ≥ the mean for aged 16–29 on memory, and performed ≥ mean − 1 *SD* for their age and education group on executive function.
**Garo-Pascual et al. (**2023)**, ** **Garo-Pascual et al. (**2024 **)**	70–85 *versus* 50–56-year-olds	**Superager**: scored ≥ the mean values for a 50–56-year-old in the memory test and within 1 *SD* of the mean or above for their age in nonmemory tests.
**Xu et al. (**2023)	65+ *versus* 65−	**Successful cognitive aging**: 65+ scores exceed 0 during the initial transformation (compared with the 65− group) and surpass >0.67 during the subsequent transformation (based on mean and *SD* values of the old age group).
**Keenan et al. (**2024 **)**	60+ *versus* 20–29	**Superager:** AVLT immediate score ≥ the mean for young adults. AVLT forgetting score ≤1 *SD* of the difference between the means for the delayed trial and trial five for young adults. And TMT-B score < mean − 1 *SD* for their age group and years of education.
***Multiple definitions***		
**Pezzoli et al. (**2024 **)**	Bottom 20% of cognitive age, top 20% of cognitive performance; 70+ *versus* 18–32	70+ years old, four types of definitions: (1) **SA-CAG**, ≤20th percentile of the cognitive age gap (cognitive-predicted age minus chronological age); (2) **SA-EM**, ≥80th percentile of age-adjusted EM composite; (3) **SA-NM**, ≥80th percentile of age-adjusted nonmemory cognition composite, and (4) **SA-CVLT**, performance comparable to young adults (18–32 years old) on the CVLT long delay free recall.

*Note*. 3MS = Modified Mini-Mental State test; ADNI-MEM = Alzheimer’s Disease Neuroimaging Initiative memory composite score; AVLT = Auditory Verbal Learning Test; CAG = cognitive age gap; CDR = clinical dementia rating; CVLT = California Verbal Learning Test; EM = episodic memory; Five-cog = five-cognitive functions; IQ = intelligent quotient; LC = latent clustering; MMSE = mini-mental state examination; NM = nonmemory; RPM = Raven’s Standard Progressive Matrices test; SA = successful cognitive aging; TMT-B = Trail-Making Test Part B.

The CCD approach utilized various thresholds, including median scores (three studies), mean scores (two studies), 10th–67th percentiles (six studies), and 0.5–1.5 *SD* above the mean (seven studies). The CCD makes full use of the cognitive aging norms and facilitates data acquisition and between-group comparisons, but it can reflect only the cognitive state at a single time point and cannot capture the dynamic nature of cognitive aging. The LTD approach was used in longitudinal studies that followed cognitive changes for 2.56–15 years. The latent clustering method is commonly used in this approach to identify “supernormal” maintainers (Chen et al., 2022; Josefsson et al., 2012; Lin et al., 2017b). Despite latent clustering, positive cognitive slopes, sustained high cognitive *Z*-scores, and maintaining normal cognition longitudinally were all incorporated in the included studies. LTD provides dynamic perspectives despite greater resource demands in longitudinal studies. GCD definitions predominantly featured “superagers,” the most distinctive SCA subgroup in our review. The Northwestern study group first defined superagers as older adults aged 80+ years with episodic memory performance at least as good as normative values for those 50- to 65-year-olds (Harrison et al., 2012), and the BRAINS program focused on a younger older adults group aged 60–80 years compared to young adults aged 18–32 years (Katsumi et al., 2021; Sun et al., 2016; Zhang et al., 2020). Other studies using the GCD have conducted various age comparisons, including 70+ *versus* 18- to 32-year-olds (Harrison et al., 2018), 60–80 *versus* 20- to 30-year-olds (Bott et al., 2017), etc. While GCD effectively demonstrates the cognitive youthfulness of SCA, significant variability in reference ages (from 16 to 60 years old) and about 30-year comparison gaps may introduce potential cohort effects.

### Genetic and epigenetic biomarkers

Genetic factors significantly influence cognitive variability, yet their role in SCA remains unclear. We synthesize studies that examine how variations in genes (DNA sequences) and their regulation (e.g., DNA methylation) may influence SCA. The apolipoprotein E (*APOE*) gene, particularly its ε4 allele (*APOE*4), represents the strongest genetic risk factor for AD (Scheltens et al., 2021). Among 20 included studies examining genetic/epigenetic biomarkers (Table 2 and Table S5), 14 investigated the *APOE*4 carrier rate. Consistent findings show lower *APOE*4 carrier rates in SCA compared to pathological cognitive aging groups (Baran et al., 2018; Josefsson et al., 2012; Lin et al., 2017b). However, most studies found no significant *APOE*4 differences between SCA and normal controls (13 studies), with one exception reporting higher *APOE*4 frequency in cognitively low stable agers than successful agers (Lin et al., 2017b). Additionally, neither *APOE*2 allele frequency nor AD polygenic hazard scores distinguished SCA individuals from normal controls (Patel et al., 2024; Spencer et al., 2022), suggesting that neurodegenerative genetic risk factors seem to minimally impact SCA.

**Table 2.** Main findings of multidomain biomarkers of successful cognitive aging.

Biomarkers	Main findings	Definition types	Publications
***Genetic and epigenetic biomarkers***			
***APOE4***	(+) *APOE4* carriers were more in low stable agers compared to successful agers.	LTD	Lin et al. (2017b)
	(−) SCA and NCA groups had no differences in *APOE4* carrying rate.	CCD, LTD, GCD	Baran et al. (2018), Bott et al. (2017), Chen et al. (2020), Dang et al. (2019a, 2019b), Dekhtyar et al. (2017), Gardener et al. (2021), Garo-Pascual et al. (2023), Gefen et al. (2015), Harrison et al. (2018), Josefsson et al. (2012), Patel et al. (2024), Pezzoli et al. (2024)
***APOE2***	(−) SCA and NCA groups had no differences in *APOE2* carrying rate.	CCD	Patel et al. (2024)
***COMT***	(+) *COMT*-met was a significant predictor of maintainers.	LTD	Josefsson et al. (2012)
**6q24**	(+) SCA was linked to the 6q24 region, including SNP rs6902875.	CCD	Barral et al. (2014)
***MAP2K3***	(+) SCA was associated with variants in the *MAP2K3* gene in three SNPs.	GCD	Huentelman et al. (2018)
**AD-PHS**	(−) There was no significant difference in the Alzheimer’s disease (AD)-PHS between Superagers and cognitively normal controls.	GCD	Spencer et al. (2022)
**DNA methylation**	(+) SCA individuals had a younger delta age (DNA methylation age minus chronological age) compared with average or accelerated decliners.	LTD	Degerman et al. (2017)
	(+) The SCA group showed significantly delayed intrinsic and extrinsic EAA than the NCA group.	CCD	Park et al. (2021)
	(+) *CEND*1 and *miR*885 were validated as having significantly different gene expressions between the SCA and NCA groups.	CCD	Park et al. (2022b)
***Biofluid biomarkers***			
**CSF Aβ**	(+) Aβ_1-42_+ were more likely to be in low stable agers compared to successful agers.	LTD	Lin et al. (2017b)
	(−) No between-group difference was found in CSF Aβ.	LTD, GCD	Chen et al. (2020), Garo-Pascual et al. (2023)
**CSF tau**	(+) Individuals with t-tau+ were more likely to appear in declining agers compared to successful agers.	LTD	Lin et al. (2017b)
	(−) No between-group difference was found in CSF tau.	LTD, GCD	Chen et al. (2020), Garo-Pascual et al. (2023)
**Plasma metabolism**	(+) A significant differential abundance of 12 metabolites was found in those with SCA relative to controls.	CCD	Mapstone et al. (2017)
**CRP**	(+) Higher CRP in cognitively intact probands was associated with a lower risk of dementia in relatives.	CCD	Silverman et al. (2012)
	(−) No between-group difference was found in CRP.	CCD	Patel et al. (2024)
**WBC**	(+) Participants from SCA families had a higher monocyte count at baseline.	CCD	Patel et al. (2024)
**IL-6**	(−) No between-group difference was found in IL-6.	CCD, GCD	Bott et al. (2017), Patel et al. (2024)
**Gut microbiome**	(+) Significant microbiome features for distinguishing superagers included Alistipes, PAC001137_g, PAC001138_g, Leuconostoc, and PAC001115_g.	GCD	Kim et al. (2024)
***Histological biomarkers***			
**AP**	(+) Superagers showed a lower frequency of AP than controls.	GCD	Gefen et al. (2015)
	(−) There were no significant differences in AP density.	CCD, GCD	Biswas et al. (2023), Gefen et al. (2021)
**NFT**	(+) Superagers showed a lower frequency of NFT than controls, especially in ERC, where controls had about threefold NFTs than superagers.	GCD	Gefen et al. (2021), Gefen et al. (2015), Nassif et al. (2022)
	(−) There were no significant differences in NFT density.	CCD	Biswas et al. (2023)
**VEN**	(+) Superagers showed a higher density of VENs than controls, especially in the cingulate regions.	GCD	Gefen et al. (2018), Gefen et al. (2015)
**ERC neuron size**	(+) Superagers had a larger soma size of layer II ERC neurons compared with controls.	GCD	Nassif et al. (2022)
**AChE-positive CPN**	(+) Superagers showed a significantly lower density of AChE-positive cortical pyramidal neurons when compared with same-age peers.	GCD	Janeczek et al. (2018)
***PET biomarkers***			
**PET Aβ**	(+) Superagers had a lower percentage of Aβ-positive or less Aβ burden compared to controls in specific regions, such as the cingulate cortex.	LTD	Arenaza-Urquijo et al. (2019), Baran et al. (2018), Harrison et al. (2024)
	(−) Superagers showed no difference in Aβ deposition compared with controls.	CCD, LTD, GCD	Borelli et al. (2021), Dang et al. (2019a, 2019b), de Souza et al. (2022), Dekhtyar et al. (2017), Dominguez et al. (2024), Gardener et al. (2021), Harrison et al. (2018), Lin et al. (2017a), Pezzoli et al. (2024)
**PET tau**	(+) Superagers had lower tau deposition compared with controls, especially in the ERC.	CCD, LTD, GCD	Harrison et al. (2024), Pezzoli et al. (2024)
	(−) Superagers showed no difference in tau deposition compared with controls.	CCD	Dominguez et al. (2024)
**Glucose metabolism**	(+) Superagers had higher glucose metabolism than controls in multiple regions, especially in the cingulate cortex.	LTD, GCD	Arenaza-Urquijo et al. (2019), Baran et al. (2018), Borelli et al. (2021)
***N*-acetyl aspartate**	(+) There was a higher total *N*-acetyl aspartate concentration in superagers than in age-matched controls.	GCD	de Godoy et al. (2021b)
***Brain structural MRI biomarkers***			
**Cortical thickness**	(+) Superagers had greater cortical thickness in multiple regions than same-age controls and even had similar cortical thickness with young controls, especially in the cingulate cortex, prefrontal cortex, etc. (Please refer to Table S10 for detailed regions.)	CCD, GCD	Dominguez et al. (2021), Fjell et al. (2006), Gefen et al. (2015), Harrison et al. (2018), Harrison et al. (2012), Katsumi et al. (2022), Pezzoli et al. (2024), Sun et al. (2016), Xu et al. (2023)
**GMV**	(+) Superagers had greater GMV than controls in multiple regions, including the medial temporal area, cingulate cortex, middle frontal cortex, etc. (Please refer to Table S10 for detailed regions.)	CCD, LTD, GCD	Bott et al. (2017), Dekhtyar et al. (2017), Gardener et al. (2021), Garo-Pascual et al. (2023), Harrison et al. (2024), Harrison et al. (2018), Klinedinst et al. (2023), Pezzoli et al. (2024), Rosano et al. (2012), Xu et al. (2023), Yang et al. (2022)
	(−) Successful older adults had smaller GMVs in the bilateral hippocampus and right parahippocampal gyrus than average older adults.	LTD	Pudas et al. (2013)
**WMH**	(+) Superagers had lower WMH volumes than controls.	GCD	Harrison et al. (2018)
**WM integrity**	(+) Superagers had higher FA and lower MD, RD, and AD than controls in multiple fibers, including the cingulum bundle, the corpus callosum, etc. (Please refer to Table S11 for detailed fibers.)	LTD, GCD	Garo-Pascual et al. (2024), Kim et al. (2020), Rosano et al. (2012), Xu et al. (2023)
	(−) There were no significant between-group differences in FA values.	LTD	Pudas et al. (2013)
**WM connectome**	(+) Superagers had stronger WM connections and greater efficiency among multiple regions, and a unique structural connectome remains stable over time in superagers relative to controls.	CCD, LTD	Chen et al. (2020), Wang and Zhang (2021), Yang et al. (2022)
***Brain functional neuroimaging biomarkers***			
**ERP**	(+) Superagers generate a larger P3 response detected by ERP to novel relative to standard stimuli than controls.	CCD	Daffner et al. (2006), Riis et al. (2008)
**EEG**	(+) Superagers showed high temporal complexity of EEG signals on a slower time scale in the frontal, parietal, and temporal lobes.	CCD	Linuma et al. (2022)
	(+) Superagers had a high betweenness centrality in the frontal region in the functional network of resting-state EEG signals.	CCD	Tobe et al. (2023)
**rsfMRI: FC**	(+) Superagers had significantly stronger or weaker FCs between the cingulate cortex and multiple regions, stronger FCs within DMN and SN, and stronger FCs of basal forebrain with putamen and insular cortex.	CCD, LTD, GCD	Borelli et al. (2021), Jia et al. (2022), Lin et al. (2017a), Zhang et al. (2020)
	(−) Superagers do not demonstrate significantly stronger FC within DMN or SN.	GCD	Keenan et al. (2024)
	(−) Within- and between-network FCs and segregation measurements of seven large-scale networks were not the differentiators between superagers and controls.	GCD	Diamond et al. (2024)
**rsfMRI: ALFF**	(+) A “Supernormal map” that differentiated superagers from controls was identified, including the right FFG, right MFG, right anterior CC, left MTG, etc.	LTD	Wang et al. (2019)
**rsfMRI: ICA**	(+) Superagers had increased FC of the right superior frontal gyrus with the independent component 6 than controls.	GCD	Borelli et al. (2021)
	(+) The DMN, SN, and language networks differentiated superagers and controls.	GCD	de Godoy et al. (2023)
	(+) Superagers had the lowest FC and possessed enhanced neural processing efficiency.	LTD	Klinedinst et al. (2023)
	(+) The SCA group had enhanced resting-state network connectivity strength in the left FPN, the anterior DMN, and the basal ganglia network.	GCD	Xu et al. (2023)
**rsfMRI: functional connectome**	(+) A functional connectome was identified, and the most discriminative nodes for predicting superagers include the precuneus, posterior CC, insular cortex, and superior, middle, and inferior frontal gyrus.	GCD	Park et al. (2022a)
**Task fMRI**	(+) Superagers showed more BOLD activation in the cingulate region than decliners.	CCD	Waiter et al. (2008)
	(+) Superagers had higher BOLD signals during encoding than average older adults, notably in the bilateral prefrontal cortex and the left hippocampus.	LTD	Pudas et al. (2013)
	(+) Superagers exhibited greater neural differentiation and neural reinstatement compared with controls.	GCD	Katsumi et al. (2021)
	(+) Superagers exhibited higher subsequent memory effects and showed additional recruitment in prefrontal clusters than controls.	LTD	Chen et al. (2022)

*Note*. Aβ = Amyloid β; AChE = acetylcholinesterase; AD = axial diffusivity; ALFF = amplitude of low-frequency fluctuation; AP = amyloid plaques; *APOE* = apolipoprotein E allele; BOLD = blood oxygenation level dependent; CC = cingulate cortex; CCD = cross-sectional comparison definition; *CEND1* = cycle exit and neuronal differentiation 1; *COMT* = catechol-*O*-methyltransferase; CPN = cortical pyramidal neuron; CRP = C-reactive protein; CSF = ­cerebrospinal fluid; DMN = default mode network; DNA = deoxyribonucleic acid; EAA = epigenetic age acceleration; EEG = electroencephalography; ERC = entorhinal cortex; ERP = event-related potential; FA = fractional anisotropy; FC = functional connectivity; FFG = fusiform gyrus; fMRI = functional magnetic resonance imaging; FPN = frontoparietal network; GCD = generational comparison definition; GMV = gray matter volume; ICA = independent components analysis; IL = interleukin; LTD = longitudinal tracking definition; *MAP2K3* = mitogen-activated protein kinase kinase 3; MD = mean diffusivity; MFG = middle frontal gyrus; *miR*885 = microRNA 885; MTG = middle temporal gyrus; NCA = normal cognitive aging; NFT = neurofibrillary tangles; PET = positron emission tomography; PHS = polygenic hazard score; RD = radial diffusivity; rsfMRI = resting-state fMRI; SCA = successful cognitive aging; SN = salience network; SNP = single-nucleotide polymorphisms; t-tau = total tau; VEN = von Economo neuron; WBC = white blood cell; WM = white matter; WMH = white matter hyperintensity.

Beyond *APOE*, several candidate genes have been identified: Josefsson et al. (2012) identified catechol-*O*-methyltransferase (*COMT*)-met genotype as predictive of cognitive maintainers in the Betula study. Whole-exome sequencing revealed associations between SCA and mitogen-activated protein kinase kinase 3 (*MAP2K3*) gene variants (Huentelman et al., 2018), and another genome-wide analysis linked exceptional cognition to the 6q24 region (Barral et al., 2014). Epigenetically, three DNA methylation studies demonstrated that SCA individuals exhibit younger biological age profiles, including younger DNA methylation age compared to average or accelerated decliners (Degerman et al., 2017), delayed epigenetic age acceleration (Park et al., 2021) and differential methylated gene expression especially in cycle exit and neuronal differentiation 1 (*CEND1*) and microRNA 885 (Park et al., 2022b).

### Biofluid biomarkers

Studies are synthesized to review measurable substances in blood, cerebrospinal fluid (CSF), or other bodily fluids that reflect brain health or pathology. Core AD biomarkers, including amyloid β_42_ (Aβ_42_), total tau protein (t-tau), and phosphorylated tau protein (p-tau) found in CSF or peripheral plasma, were examined in three studies to assess neuropathological resistance in SCA (Table 2 and Table S6). While Aβ_42_+ and t-tau+ individuals were more likely to be classified as cognitively low stable and declining agers than successful agers (Lin et al., 2017b), no significant difference in CSF Aβ/p-tau ratio was observed between SCA and average agers (Chen et al., 2020), nor in Aβ_42_/Aβ_40_ ratio, t-tau, p-tau, or p-tau/Aβ_42_ ratio (Garo-Pascual et al., 2023). Three of the included studies examined peripheral inflammatory markers with mixed findings. Higher C-reactive protein (CRP) levels were found associated with reduced familial dementia risk (Silverman et al., 2012), while resilient agers showed lower levels of interleukin-6 (IL-6) than did declining agers but not average agers (Bott et al., 2017), and SCA group had a marginally higher monocyte white blood cells counts and no significant difference in CRP or IL-6 levels compared with the control group (Patel et al., 2024). Additionally, metabolomic analyses identified 12 plasma metabolites distinguishing SCA and controls (area under the curve = 0.89) (Mapstone et al., 2017), and significant differences in the gut microbiome between superagers and typical agers were found with several selected microbial features (Kim et al., 2024). These emerging biomarkers highlight novel pathways in cognitive resilience.

### Histological biomarkers

Six histological studies examined whether SCA reflects resistance to AD-related neuropathology or distinct neurophysiological mechanisms (Table 2 and Table S7), and these biomarkers are derived from postmortem brain tissue analysis, revealing microscopic changes linked to cognitive aging. The Northwestern University superaging group conducted a series of histological investigations revealing that superagers exhibited approximately one-third fewer AD-type neurofibrillary tangles (NFTs) in the entorhinal cortex (ERC) (Gefen et al., 2015, 2021; Nassif et al., 2022), and demonstrated larger soma sizes of ERC neurons (Nassif et al., 2022) compared to normal controls and even younger controls. In addition, superagers had fewer amyloid plaques in the anterior cingulate regions (Gefen et al., 2015). However, the 90+ study found no association between AD neuropathological changes and SCA (Biswas et al., 2023).

In addition to the findings for AD-related pathology, histological studies have identified several distinctive neurobiological features associated with SCA (Table 2 and Table S7). Von Economo neurons (VENs), specialized large bipolar neurons unique to humans and great apes, were found to be significantly denser in superagers compared to controls, particularly in anterior cingulate regions (Gefen et al., 2015, 2018). Additionally, an investigation on cholinergic pathways revealed that superagers exhibited reduced staining intensity and density of ­acetylcholinesterase (AChE)-positive cortical pyramidal ­neurons than controls (Janeczek et al., 2018), suggesting enhanced cholinergic function. This finding aligns with evidence of SCA individuals showing less cholinesterase-associated neuropathology in prefrontal and hippocampal regions compared to AD patients (Maxwell et al., 2022). Collectively, these findings suggest that SCA may involve both resistance to certain age-related neuropathological changes and the preservation of distinct neurobiological features.

### PET biomarkers

Positron emission tomography imaging studies track molecular activity in the living brain to identify patterns associated with superaging. Fourteen PET studies examined various pathophysiological markers in SCA, including Aβ, tau, glucose metabolism, and *N*-acetyl aspartate (Table 2 and Table S8). Aβ deposition was investigated in 13 studies, and most of the results (10 studies) consistently revealed no significant difference in Aβ burden between SCA and normal control groups, aligning with the aforementioned biofluid and histological findings. Only three studies documented significant group differences: reduced regional Aβ deposition in the cingulate isthmus of SCA individuals than normal controls (Baran et al., 2018) or *versus* cognitive decliners, not normal controls (Arenaza-Urquijo et al., 2019; Harrison et al., 2024). In addition, longitudinal assessments suggested Aβ status, rather than SCA classification, influenced brain atrophy and cognition (Dang et al., 2019a, 2019b), and no difference was found in the rates of change in AD-related neuropathological biomarkers between maintainers and decliners (Harrison et al., 2024). Tau PET results were mixed: two studies found that SCA individuals presented lower levels of tau accumulation in the ERC (Harrison et al., 2024; Pezzoli et al., 2024), aligning with histological findings, while one showed no difference in tau deposition (Dominguez et al., 2024).

Cerebral glucose metabolism is one of the physiological indicators of cognitive function. Three [^18^F]-fluorodeoxyglucose-PET studies demonstrated significantly higher metabolism in the SCA group than controls in the cingulate cortex, anterior temporal pole, hippocampus, and several frontal and temporal regions (Arenaza-Urquijo et al., 2019; Baran et al., 2018; Borelli et al., 2021). Notably, a “resilience signature” was identified as predictive of cognitive stability independent of Aβ status (Arenaza-Urquijo et al., 2019). Another brain metabolic signature was found using proton magnetic resonance spectroscopy (^1^H-MRS), showing superagers exhibit higher *N*-acetyl aspartate concentrations in the posterior cingulate cortex (de Godoy et al., 2021b), also suggesting enhanced neuronal viability in SCA individuals.

### Brain structural MRI biomarkers

**Figure 3.**
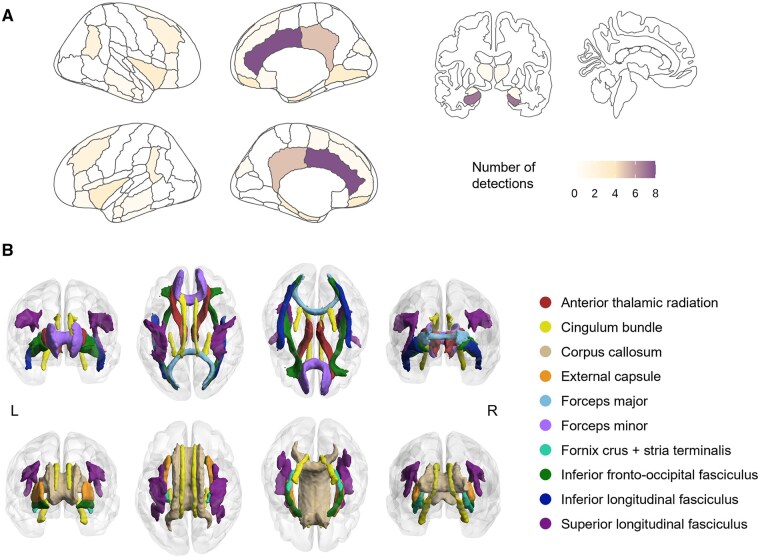
Summarized brain signatures of regions showing significant structural preservation in successful cognitive aging (SCA) individuals. (A) Gray matter signature: The cortical regions are mapped in the left panel, and the subcortical regions are mapped in the right panel. The color change from yellow to purple indicates an increasing number of findings with SCA individuals having significantly better gray matter preservation than normal controls in included studies; this figure is depicted via R software with the “ggseg” package (https://ggseg.github.io/ggseg/). (B) White matter signature: Tracts presented in the upper panel are extracted from the Johns Hopkins University white matter tractography atlas (including 20 tracts), and tracts presented in the bottom panel are extracted from the ICBM-DTI-81 white matter labels atlas (including 48 tracts).

Structural MRI findings from 22 studies measured physical brain characteristics, including cerebral thickness (CT), GM volume, white matter hyperintensity (WMH), etc., and revealed consistent brain preservation patterns in SCA (Table 2 and Table S9). One typical brain region involved in SCA’s structural preservation was the cingulate cortex, which was detected 14 times in nine studies. Initial observations by Fjell et al. (2006) demonstrated superior CT in high cognitive performers compared to average peers, with posterior cingulate regions even exceeding young controls. Subsequent research confirmed this cingulate preservation across its anatomical subdivisions, including the anterior cingulate cortex (eight studies), posterior cingulate cortex (four studies), cingulate gyrus isthmus, and paracingulate cortex. The hippocampus emerged as another key region of structural preservation of SCA, with seven studies reporting larger volumes compared to controls, extending to its neighboring structures in the medial temporal lobe (MTL), such as the parahippocampal gyrus and the ERC (Garo-Pascual et al., 2023; Pezzoli et al., 2024; Rosano et al., 2012). Preservations of frontal structures are also relatively common in SCA studies (Fjell et al., 2006; Harrison et al., 2018; Pezzoli et al., 2024; Sun et al., 2016), and were further emphasized in the “frontal preservation, temporal impairment (FPTI)” hypothesis (Yang et al., 2022), claiming that frontal preservation is one of the specific brain substrates of SCA. Subcortical structures, including the insula, basal forebrain, amygdala, and thalamus, also demonstrated GM advantages in SCA, and many fewer results were distributed in middle–posterior brain regions. Evidence for the longitudinal brain atrophy rate of SCA individuals is controversial, with some reporting slower hippocampal decline (Garo-Pascual et al., 2023; Harrison et al., 2024), while others found no group differences (Gardener et al., 2021; Harrison et al., 2018). A representative result regarding GM substrates of SCA was defined by Sun et al. (2016) as a “superaging signature.” Based on prior work, we summarize an updated GM preserved signature of SCA as shown in Figure 3A and Table S10.

White matter investigations, though fewer in number, revealed important microstructural findings (Table 2 and Table S9). SCA individuals exhibited lower WMH burden (Harrison et al., 2018) and superior integrity (higher fractional anisotropy or lower mean diffusivity) in key tracts, including the cingulate cortex, the corpus callosum, and the superior longitudinal fasciculus (Kim et al., 2020; Rosano et al., 2012). A “frontal preservation”-type distribution of SCA’s WM advantage was found: an anteroposterior gradient with greater group differences in anterior tracts (Garo-Pascual et al., 2024). WM fibers that showed significantly greater integrity in SCA are summarized in Figure 3B and Table S11. WM connectome analyses revealed distinctive network properties in SCA, including stable structural connectome contributing to the prediction of pathology resistance (Chen et al., 2020), enhanced frontal connections (Wang & Zhang, 2021), and superior topological efficiency in frontal–basal ganglia regions (Yang et al., 2022), suggesting that preserved WM connectivity complements regional GM advantages in SCA, especially in frontal regions.

### Brain functional neuroimaging biomarkers

Using functional MRI (fMRI) and related techniques, SCA individuals were found with unique advantages in dynamic brain functional activity that are strongly associated with their superior cognition (Table 2 and Table S12). Resting-state fMRI studies yield mixed functional connectivity (FC) findings. Four studies reported positive results indicating that FC differentiates between SCA individuals and average cognitive agers in the cingulate cortex (Borelli et al., 2021; Lin et al., 2017a), within both the default mode network (DMN) and salience network (SN) (Zhang et al., 2020), and between basal forebrain with putamen and insular cortex (Jia et al., 2022). In contrast, the other two studies find no significant FC differences in network level (Diamond et al., 2024; Keenan et al., 2024). Independent component analysis studies identified key differentiating networks (the DMN, SN, frontoparietal network, basal ganglia network, and language network) (Borelli et al., 2021; de Godoy et al., 2023; Xu et al., 2023), and proposed an enhanced neural efficiency hypothesis in SCA individuals (Klinedinst et al., 2023), and a machine learning study revealed discriminative nodes in cingulate and frontal regions for SCA prediction (Park et al., 2022a). Benchmarking the “superaging signature” proposed by brain structural studies, a “supernormal map” was defined as significantly differentiating SCA individuals and normal controls longitudinally (Wang et al., 2019), and regions in this map are similar to the structurally preserved regions summarized by this review (Figure 3A and Table S10).

Electroencephalography/ERP studies found a larger anterior P3 response to novel stimuli (Daffner et al., 2006; Riis et al., 2008), and greater temporal synchronicity, complexity, and interaction of brain activities (Linuma et al., 2022; Tobe et al., 2023) in SCA, both suggesting frontal compensatory. Task-based fMRI studies have provided further evidence of SCA’s frontal mechanism. SCA individuals showed greater activation in the prefrontal and hippocampal cortex (Pudas et al., 2013), greater neural differentiation during encoding, and greater neural reinstatement between encoding and retrieval (Katsumi et al., 2021), all of which were correlated with their memory performance. In addition, Chen et al. (2022) designed a subsequent memory paradigm and found that successful agers both exhibited greater subsequent memory effects on task-related regions and had compensatory frontal recruitment. These findings collectively highlight the frontal regions’ crucial role in cognitive preservation, complementing structural observations.

### Integration of SCA biomarkers

**Figure 4.**
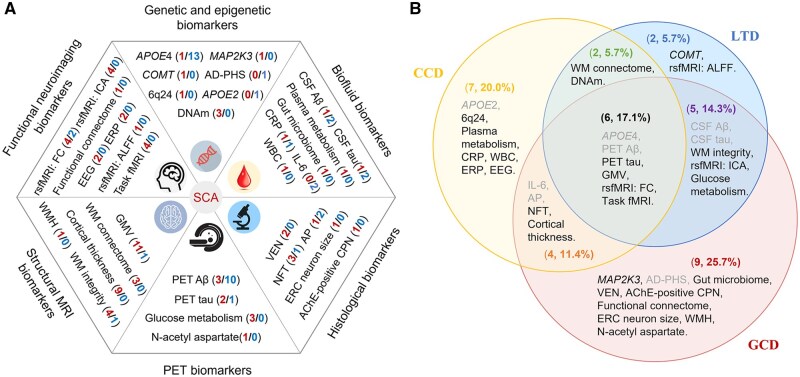
Integration of biomarker findings of successful cognitive aging based on six biomarker domains and three types of definitions. (A) A hexagonal diagram showing all biomarkers discussed in this review by six domains. The numbers in parentheses indicate the number of studies with positive findings (in red color)/the number of studies with negative findings (in blue color); (B) A Venn diagram dividing all biomarkers based on the definitions these studies used. Biomarkers in gray color were those with over half of the related studies showing negative findings. AChE = acetylcholinesterase; AD-PHS = Alzheimer’s disease polygenic hazard score; ALFF = amplitude of low-frequency fluctuation; AP = amyloid plaques; *APOE* = apolipoprotein E; Aβ = amyloid β; CCD = cross-sectional comparison definition; *COMT* = catechol-O-methyltransferase; CPN = cortical pyramidal neuron; CRP = C-reactive protein; CSF = cerebrospinal fluid; DNAm = deoxyribonucleic acid methylation; EEG = electroencephalography; ERC = entorhinal cortex; ERP = event-related potential; FC = functional connectivity; fMRI = functional magnetic resonance imaging; GCD = generational comparison definition; GMV = gray matter volume; ICA = independent components analysis; IL = interleukin; LTD = longitudinal tracking definition; *MAP2K3* = mitogen-activated protein kinase kinase 3; MRI = magnetic resonance imaging; MTL = medial temporal lobe; NFT = neurofibrillary tangles; PET = positron emission tomography; rsfMRI = resting-state magnetic resonance imaging; SCA = successful cognitive aging; VEN = von Economo neuron; WBC = white blood cell; WM = white matter; WMH = white matter hyperintensity.

All identified biomarkers are integrated based on the six-domain biomarker classification (Figure 4A) and the three-type definitional framework (Figure 4B). The most frequently studied biomarkers (examined in more than 10 studies) include *APOE*4, PET Aβ, and brain GM morphology, with the first two being found with mainly negative findings (i.e., minimal differences between SCA individuals and normal controls on *APOE*4 carrier rate or PET Aβ deposition). In contrast, consistent positive findings emerged from DNA methylation, VEN density, and PET glucose metabolism, along with various neuroimaging findings across multiple brain regions. From the perspective of definitional differences, only 6 of 35 biomarkers (17.1%) were examined across all three definitional types, while over half were investigated using just one definition type—often in single studies. Among the 62 included studies, 19 (30.6%) studies employed multidomain approaches (Table S13), yet only two (Gardener et al., 2021; Pudas et al., 2013) of them explored cross-domain interactions. These findings suggest that while current research has established a preliminary SCA biomarker framework, critical knowledge gaps remain. Future studies should particularly address: (1) definition-dependent consistency in biomarker findings, and (2) the synergistic effects of cross-domain biomarkers on ­cognitive outcomes.

## Discussion

This systematic review synthesizes evidence from 62 studies examining biomarkers of SCA, addressing a critical gap in understanding the underlying mechanisms of exceptional cognition in old age. We identified a three-type definitional framework and analyzed six biomarker domains (genetic/epigenetic, biofluid, histological, PET, structural MRI, and functional neuroimaging biomarkers), revealing both consistent findings and important divergences in this emerging field.

This review highlights substantial heterogeneity in SCA definitions, identifying 14 diverse terms and 34 distinct definitions across included studies. This definitional variability poses challenges for comparative research, though the use of validated neuropsychological tests provides some standardization. We identified a “three-type” framework for SCA definitions: CCD, LTD, and GCD. Based on prior work, we propose several possible solutions to enhance definitional consistency, including: (1) prioritizing longitudinal designs (Nyberg, 2024) and data-driven methods such as latent clustering; (2) prioritizing episodic memory and executive function as assessed cognitive domains, and using TMT-B and Rey AVLT as specific cognitive tests; (3) applying the “three-type” framework of SCA definitions in the same study and selecting the overlapped sample of different definitions based on big data; and (4) expanding cultural diversity in study samples and conducting cross-cultural comparison studies.

Findings in this review suggest that the core mechanism of SCA differs from that of AD-related pathological cognitive aging. Aβ positivity is considered the pathological “gold standard” for AD, and high levels of *APOE*4 carrying rate and tau deposition are also characteristics of neurodegeneration (Jack et al., 2024). The relationship between *APOE*4 and SCA appears minimal, with only one of 14 studies reporting a significant difference between successful agers and low-stable agers, which were driven by latent classification analysis and were not proper normal controls (Lin et al., 2017b). Similarly, most studies found no Aβ differences between SCA individuals and normal controls (10 of 13 PET studies, two of three biofluid studies, and two of three histological studies), with exceptions showing reduced Aβ burden of the SCA group specifically in the cingulate cortex (Baran et al., 2018; Gefen et al., 2015), or less Aβ pathology in SCA individuals than cognitive decliners or low-stable agers, neither of which were proper normal controls (Arenaza-Urquijo et al., 2019; Harrison et al., 2024; Lin et al., 2017b). Controversial results were obtained for tau or NFTs in SCA studies: 6 of 10 studies reported significantly lower levels of tau in the CSF, postmortem brain issues, or PET images in SCA individuals, with a specific focus on the ERC, while the other four studies found no difference. Definitional differences and measurement techniques may account for these divergent findings, and more validating studies are needed in this field. It is worth noting that the aforementioned findings imply SCA individuals’ regional resistance to AD-related pathologies (Aβ and tau) in the cingulate and the medial temporal cortex, which aligns with their structural preservation.

Some consistently positive results may represent the specific resilience of SCA individuals. Epigenetic studies revealed that SCA individuals have a young DNA methylation age (Degerman et al., 2017; Park et al., 2021, 2022b), which tends to be a valuable biomarker for identifying SCA. Based on these positive results and the recent progress of aging clock studies, other biological age prediction models, such as brain age (Seitz-Holland et al., 2024), may also play an important role in identifying SCA. Another positive and consistent biomarker is high glucose metabolism in SCA individuals (Arenaza-Urquijo et al., 2019; Baran et al., 2018; Borelli et al., 2021). These studies emphasized the cingulate cortex as a crucial region and that glucose metabolism is independent of the Aβ burden. Having a healthier metabolic profile was also identified as a biofluid biomarker of SCA (Mapstone et al., 2017). The cingulate cortex was also highlighted in the findings of histological biomarkers with a high VEN density (Gefen et al., 2015, 2018), whose biological meaning has been interpreted as contributing to individual differences in general intelligence (*g*) by rapidly inducing the coherence of neuronal oscillations (Bruton, 2021), which needs further exploration in the aging process. Other potential biomarkers, including *COMT*, *MAP2K3*, ERC neuron size, AChE level, WMH, etc., require further validation due to limited replication.

A robust finding emerging from our cross-domain synthesis is the identification of a characteristic “cingulate gyrus–MTL–frontal cortex” signature, which may serve as a potential unifying framework for understanding the brain substrate of SCA. The cingulate cortex demonstrates convergent preservation across multiple biomarker domains in SCA individuals, showing lower Aβ deposition (Baran et al., 2018; Gefen et al., 2015), higher VEN density (Gefen et al., 2015, 2018), enhanced glucose metabolism (Arenaza-Urquijo et al., 2019; Baran et al., 2018; Borelli et al., 2021), and elevated *N*-acetyl aspartate concentrations (de Godoy et al., 2021b). The MTL, particularly the hippocampus, emerges as a pivotal substrate for both pathological and successful cognitive aging, likely due to its central role in episodic memory (Moscovitch et al., 2016), the most marked cognitive domain estimated in cognitive aging. The frontal components of this signature are particularly noteworthy; multiple neuroimaging signatures of SCA emphasized frontal regions (Sun et al., 2016; Wang et al., 2019; Zhang et al., 2020), and the recently proposed FPTI hypothesis (Yang et al., 2022) established a pivotal role of the frontal structure preservation in SCA. Moreover, the frontal maintenance and compensation mechanisms of brain function are commonly identified (Chen et al., 2022; Pudas et al., 2013; Vidal-Pineiro et al., 2019), suggesting that multiple protective mechanisms may converge in frontal regions. This tripartite signature aligns remarkably well with several established models of brain aging: (1) It encompasses key nodes of the DMN whose integrity is crucial for memory function and cognitive aging (Sun et al., 2016); (2) it reflects the frontal-predominant vulnerability and compensation predicted by the frontal aging hypothesis (West, 1996), the posterior–anterior shift in aging (Davis et al., 2008), and the scaffolding theory of aging and cognition (Reuter-Lorenz & Park, 2014). In summary, the “cingulate gyrus–MTL–frontal cortex” brain signature of SCA proposed by this review is supported by converging evidence, providing a testable model for future SCA research.

There are six domains of biomarkers that are synthesized and compared in this current review. Each biomarker category presents unique strengths and limitations: Genetic biomarkers offer stable early-risk assessment but lack dynamic information and typically unchangeable; biofluid biomarkers (e.g., plasma, saliva, urine) offer significant practical advantages as minimally invasive and cost-effective tools yet peripheral measures may not fully reflect brain pathology; histological biomarkers provide gold-standard validation but require postmortem tissue; PET imaging delivers molecular specificity at a high cost; structural MRI balances spatial precision with clinical accessibility; functional neuroimaging reveals neural mechanisms but shows state-dependent variability. For implementation, biofluid biomarkers and structural MRI currently may show the best feasibility-profile balance by now. After synthesizing, we found that most included studies treat biomarkers additively rather than interactively, and the cross-domain relationships between biomarkers are still unclear. We would like to emphasize that multimodal approaches used to explore SCA biomarkers and investigations on uncovering cross-domain interactions may optimize future research and applications (Bao et al., 2023), and emerging mobile health technologies and AI models may integrate multimodal data for better cognitive predictions and support (Czaja et al., 2025).

This systematic review has several limitations. First, the comparability of findings may be affected by differing SCA definitions, though most studies used validated neuropsychological tests and age-matched controls for reliable reference standards. Second, some studies had a small sample of SCA individuals, potentially limiting statistical power, and over half of the included studies were conducted in North America, which may reduce cultural generalizability. Third, due to methodological diversity across domains and the small sample sizes of some studies, the present study did not conduct a meta-analysis; future studies could include meta-analyses of SCA biomarkers as more findings become available.

## Conclusions

This systematic review synthesizes evidence from 62 studies to characterize SCA through three definitional approaches and six biomarker domains. The overall profile of biomarkers shows that SCA represents an active neurobiological process marked by unique resilience mechanisms, rather than merely reflecting resistance to typical age-related pathologies (e.g., Aβ/tau deposition). Key findings demonstrate that SCA individuals have specific resilience mechanisms, as represented by their youthful epigenetic age, high VEN density, efficient glucose metabolism, and structural and functional integrity of critical brain networks. By summarizing neuroimaging studies, superior brain reserve, maintenance, and compensation in SCA individuals were found in brain regions centered on a “cingulate gyrus–MTL–frontal cortex” signature. The findings suggest that the aging process involves not only a decline and loss but also a process of maintenance and adaptation, emphasizing the importance of shifting research paradigms to better understand and optimize neural resilience capacities. We advocate for more studies on SCA’s biological mechanisms and interventions targeting clear resilience pathways in the future. Such work could ultimately enable more individuals to achieve healthy, graceful aging.

## Supplementary Material

gnaf277_Supplementary_Data

## Data Availability

This systematic review is registered at PROSPERO under the following identification number: CRD42024578134 (https://www.crd.york.ac.uk/PROSPERO/view/CRD42024578134). All data supporting the findings of this study are available within the paper and its online supplementary material.
