# Supplementary material for: What characterizes the exceptional cognition of superagers? A systematic review of multidomain biomarkers of successful cognitive aging
Source: Gerontologist. 2025 Nov 24;66(4):gnaf277. doi: 10.1093/geront/gnaf277 (PMC13017228; doi:10.1093/geront/gnaf277)
Supplement: gnaf277_Supplementary_Data [file gnaf277_supplementary_data.pdf]

What Characterizes the Exceptional Cognition of Superagers? A Systematic Review of Multidomain Biomarkers of Successful Cognitive Aging

SUPPLEMENTAL MATERIAL

Yiru Yang, Ph.D.[1*], Xiaolei Li, B.Sc.[1], Shudan Gao, Ph.D.[2], Yuanxu Gao, Ph.D.[3]

[1]School of Nursing and Rehabilitation, Cheeloo College of Medicine, Shandong University, Jinan, Shandong, 250012, China.

[2]Shandong Provincial Key Laboratory of Brain Science and Mental Health, Faculty of Psychology, Shandong Normal University, Jinan, 250358, China.

[3]Institute for AI in Medicine and Faculty of Medicine, Macau University of Science and Technology, Macau, 999078, China.

[*]Correspondence: Dr. Yiru Yang, Email: yangyiru@email.sdu.edu.cn; Phone/Fax number: +86 531 88382268; Full postal address: School of Nursing and Rehabilitation, Cheeloo College of Medicine, Shandong University, No.44 Wenhuaxi Road, Lixia District, Jinan, Shandong, 250012, China. ORCID: 0000-0003-1190-7540.

Content

Table S1. Included studies in the current review.

Table S2. Characteristics of the studies included in this systematic review.

Table S3. Quality assessment of included studies.

Table S4. Cognitive tests used for SCA definition in the included articles.

Table S5. Genetic and epigenetic biomarkers of successful cognitive aging.

Table S6. Biofluid biomarkers of successful cognitive aging.

Table S7. Histological biomarkers of successful cognitive aging.

Table S8. PET biomarkers of successful cognitive aging.

Table S9. Brain structural MRI biomarkers of successful cognitive aging.

Table S10. Preserved brain gray matter structures of SCA individuals revealed by studies shown in Table S9.

Table S11. Preserved brain white matter structures of SCA individuals revealed by studies shown in Table S9.

Table S12. Brain functional neuroimaging biomarkers of successful cognitive aging.

Table S13. Cross-domain relationships in included studies that explored multidomain biomarkers.

Table S1. Included studies in the current review.

Number	Article	Title	Journal
1	(Fjell et al., 2006)	Selective increase of cortical thickness in high-performing elderly—structural indices of optimal cognitive aging	NeuroImage
2	(Daffner et al., 2006)	Age-related differences in attention to novelty among cognitively high performing adults	Biol Psychol
3	(Riis et al., 2008)	Compensatory neural activity distinguishes different patterns of normal cognitive aging	NeuroImage
4	(Waiter et al., 2008)	Is retaining the youthful functional anatomy underlying speed of information processing a signature of successful cognitive ageing? An event-related fMRI study of inspection time performance	NeuroImage
5	(Rosano et al., 2012)	Neuroimaging differences between older adults with maintained versus declining cognition over a 10-year period	NeuroImage
6	(Silverman et al., 2012)	C-reactive protein and familial risk for dementia: a phenotype for successful cognitive aging	Neurology
7	(Harrison et al., 2012)	Superior memory and higher cortical volumes in unusually successful cognitive aging	J Int Neuropsych Soc
8	(Josefsson et al., 2012)	Genetic and lifestyle predictors of 15-year longitudinal change in episodic memory	J Am Geriatr Soc
9	(Pudas et al., 2013)	Brain characteristics of individuals resisting age-related cognitive decline over two decades	J Neurosci
10	(Barral et al., 2014)	Common genetic variants on 6q24 associated with exceptional episodic memory performance in the elderly	JAMA Neurol
11	(Gefen et al., 2015)	Morphometric and histologic substrates of cingulate integrity in elders with exceptional memory capacity	J Neurosci
12	(Sun et al., 2016)	Youthful brains in older adults: preserved neuroanatomy in the default mode and salience networks contributes to youthful memory in superaging	J Neurosci
13	(Lin, Ren, et al., 2017)	The cingulate cortex of older adults with excellent memory capacity	Cortex
14	(Bott et al., 2017)	Youthful processing speed in older adults: genetic, biological, and behavioral predictors of cognitive processing speed trajectories in aging	Front Aging Neurosci
15	(Lin, Wang, et al., 2017)	Identification of successful cognitive aging in the Alzheimer's disease neuroimaging	J Alzheimers Dis

		initiative study	
16	(Mapstone et al., 2017)	What success can teach us about failure: the plasma metabolome of older adults with superior memory and lessons for Alzheimer’s disease	Neurobiol Aging
17	(Dekhtyar et al., 2017)	Neuroimaging markers associated with maintenance of optimal memory performance in late-life	Neuropsychologia
18	(Degerman et al., 2017)	Maintained memory in aging is associated with young epigenetic age	Neurobiol Aging
19	(Gefen et al., 2018)	Von Economo neurons of the anterior cingulate across the lifespan and in Alzheimer's disease	Cortex
20	(Janeczek et al., 2018)	Variations in acetylcholinesterase activity within human cortical pyramidal neurons across age and cognitive trajectories	Cereb Cortex
21	(Huentelman et al., 2018)	Associations of MAP2K3 gene variants with superior memory in superagers.	Front Aging Neurosci
22	(Harrison et al., 2018)	Brain morphology, cognition, and beta-amyloid in older adults with superior memory performance.	Neurobiol Aging
23	(Baran et al., 2018)	Amyloid and FDG PET of successful cognitive aging: global and cingulate-specific differences.	J Alzheimers Dis
24	(Wang et al., 2019)	Longitudinal functional brain mapping in supernormals	Cereb Cortex
25	(Arenaza-Urquijo et al., 2019)	The metabolic brain signature of cognitive resilience in the 80+: beyond Alzheimer pathologies	Brain
26	(Dang, Harrington, et al., 2019)	Superior memory reduces 8-year risk of mild cognitive impairment and dementia but not amyloid beta-associated cognitive decline in older adults	Arch Clin Neuropsych
27	(Dang, Yassi, et al., 2019)	Rates of age- and amyloid beta-associated cortical atrophy in older adults with superior memory performance	Alzheimers Dement DADM
28	(Zhang et al., 2020)	Stronger functional connectivity in the default node and salience networks is associated with youthful memory in superaging	Cereb Cortex
29	(Chen et al., 2020)	Cognitively supernormal older adults maintain a unique structural connectome that is resistant to Alzheimer's pathology	NeuroImage-Clin
30	(Kim et al., 2020)	White matter integrity is associated with the amount of physical activity in older adults with super-aging	Front Aging Neurosci

31	(Borelli et al., 2021)	Increased glucose activity in subgenual anterior cingulate and hippocampus of high performing older adults, despite amyloid burden	J Alzheimers Dis
32	(Gardener et al., 2021)	Longitudinal trajectories in cortical thickness and volume atrophy: superior cognitive performance does not protect against brain atrophy in older adults	J Alzheimers Dis
33	(Gefen et al., 2021)	Paucity of entorhinal cortex pathology of the Alzheimer's type in superagers with superior memory performance	Cereb Cortex
34	(Park et al., 2021)	Accelerated epigenetic age in normal cognitive aging of Korean community-dwelling older adults	Biol Res Nurs
35	(Wang & Zhang, 2021)	Classification of longitudinal brain networks with an application to understanding superior aging	Stat
36	(de Godoy et al., 2021)	The brain metabolic signature in superagers using in vivo 1H-MRS: a pilot study	Am J Neuroradiol
37	(Katsumi et al., 2021)	Greater neural differentiation in the ventral visual cortex is associated with youthful memory in SuperAging	Cereb Cortex
38	(Dominguez et al., 2021)	Regional cortical thickness predicts top cognitive performance in the elderly	Front Aging Neurosci
39	(J. Park et al., 2022)	CEND1 and miR885 methylation changes associated with successful cognitive aging in community-dwelling older adults	Exp Gerontol
40	(Jia et al., 2022)	The functional connectivity of basal forebrain is associated with superior memory performance in older adults: a case-control study	BMC Geriatr
41	(Katsumi et al., 2022)	Structural integrity of the anterior mid-cingulate cortex contributes to resilience to delirium in SuperAging	Brain Commun
42	(de Souza et al., 2022)	Amyloid-β PET classification on cognitive aging stages using the centiloid scale	Mol Imaging Biol
43	(Chen et al., 2022)	Functional activation features of memory in successful agers across the adult lifespan	NeuroImage
44	(Spencer et al., 2022)	Alzheimer's polygenic hazard score in SuperAgers: SuperGenes or SuperResilience?	Alzheimers Dement
45	(Linuma et al., 2022)	Enhanced temporal complexity of EEG signals in older individuals with high cognitive functions	Front Neurosci
46	(C.-h. Park et al., 2022)	Predicting superagers by machine learning classification based on the functional brain connectome using resting-state functional magnetic resonance imaging	Cereb Cortex
47	(Nassif et al., 2022)	Integrity of neuronal size in the entorhinal cortex is a biological substrate of	J Neurosci

		exceptional cognitive aging	
48	(Yang et al., 2022)	Successful or pathological cognitive aging? Converging into a "frontal preservation, temporal impairment (FPTI)" hypothesis	Sci Bull
49	(Biswas et al., 2023)	Superior global cognition in oldest-old is associated with resistance to neurodegenerative pathologies: results from the 90+ Study	J Alzheimers Dis
50	(de Godoy et al., 2023)	Phenotyping superagers using resting-state fMRI	Am J Neuroradiol
51	(Tobe et al., 2023)	Hub structure in functional network of EEG signals supporting high cognitive functions in older individuals	Front Aging Neurosci
52	(Garo-Pascual et al., 2023)	Brain structure and phenotypic profile of superagers compared with age-matched older adults: a longitudinal analysis from the Vallecas project	Lancet Health Longev
53	(Klinedinst et al., 2023)	Exploring the secrets of super-aging: a UK Biobank study on brain health and cognitive function	GeroScience
54	(Xu et al., 2023)	Exploring successful cognitive aging: insights regarding brain structure, function, and demographics	Brain Sci
55	(Pezzoli et al., 2023)	Successful cognitive aging is associated with thicker anterior cingulate cortex and lower tau deposition compared to typical aging	Alzheimers Dement
56	(Patel et al., 2024)	Systemic inflammation in relation to exceptional memory in the Long-Life Family Study (LLFS)	Brain Behav Immun-Hl
57	(Keenan et al., 2024)	Intrinsic functional connectivity strength of SuperAgers in the default mode and salience networks: Insights from ADNI	Aging Brain
58	(Garo-Pascual et al., 2024)	Superagers resist typical age-related white matter structural changes	J Neurosci
59	(Harrison et al., 2024)	Cognitive trajectories and Alzheimer disease biomarkers: from successful cognitive aging to clinical impairment	Ann Neurol
60	(Diamond et al., 2024)	SuperAging functional connectomics from resting-state functional MRI	Brain Commun
61	(Dominguez et al., 2024)	Resilience to AD pathology in top cognitive performers	Front Aging Neurosci
62	(Kim et al., 2024)	Predicting superagers: a machine learning approach utilizing gut microbiome features	Front Aging Neurosci

Note. the number is ordered by the publication date and is consistent with other tables.

Table S2. Characteristics of the studies included in this systematic review.

Num.	Publication	Terms of SCA	SCA Sample size (Women/Men)	Country	Database
1	(Fjell et al., 2006)	High fluid/executive function	NA (NA)	Norway	Community dwellers
2	(Daffner et al., 2006)	Cognitively high performer	16 (9/7)	USA	Community dwellers
3	(Riis et al., 2008)	Cognitively high performer	16 (9/7)	USA	Community dwellers
4	(Waiter et al., 2008)	Cognitive sustainer	25 (11/14)	UK	The ABC 1936
5	(Rosano et al., 2012)	Maintainers	153 (90/63)	USA	The Health ABC study
6	(Silverman et al., 2012)	SCA	277 (0/277)	USA	Male veteran outpatients at the JJP-VAMC
7	(Harrison et al., 2012)	Superager	12 (NA)	USA	The Northwestern's AD Center, the ADNI
8	(Josefsson et al., 2012)	Maintainers	285 (NA)	Sweden	The Betula study
9	(Pudas et al., 2013)	Maintainers	51 (38/13)	Sweden	The Betula study
10	(Barral et al., 2014)	Exceptional episodic memory	467 (NA)	USA	The LLFS, the NIA-LOAD, the ADNI, the ADGC, and the WHICAP
11	(Gefen et al., 2015)	Superager	36 (26/10)	USA	The Northwestern's AD Center, the ADNI
12	(Sun et al., 2016)	Superager	17 (12/5)	USA	The BRAINS program
13	(Lin, Ren, et al., 2017)	Supernormal	9 (8/1)	USA	The ADNI
14	(Bott et al., 2017)	Resilient-ager	17 (7/10)	USA	Cohorts at the UCSF Memory and Aging Center
15	(Lin, Wang, et al., 2017)	Supernormal	144 (NA)	USA	The ADNI
16	(Mapstone et al., 2017)	Supernormal	41 (21/20)	USA	The R/OCAS
17	(Dekhtyar et al., 2017)	Optimal Memory Performer	25 (16/9)	USA	The HABS
18	(Degerman et al., 2017)	Maintainers	16 (8/8)	Sweden	The Betula study
19	(Gefen et al., 2018)	Superager	5 (5/0)	USA	The Northwestern's AD Center

20	(Janeczek et al., 2018)	Superager	5 (5/0)	USA	The Northwestern's AD Center
21	(Huentelman et al., 2018)	Superager	56 (39/17)	USA	The NSAP and the ADNI
22	(Harrison et al., 2018)	Successful ager	26 (23/3)	USA	The BACS
23	(Baran et al., 2018)	Supernormal	122 (72/50)	USA	The ADNI
24	(Wang et al., 2019)	Supernormal	13 (8/5)	USA	The ADNI
25	(Arenaza-Urquijo et al., 2019)	Cognitively stable 80+	192 (90/102)	USA	The MCSA and the ADNI
26	(Dang, Harrington, et al., 2019)	Superager	179 (96/83)	Australia	The AIBL study
27	(Dang, Yassi, et al., 2019)	Superager	172 (96/76)	Australia	The AIBL study
28	(Zhang et al., 2020)	Superager	17 (12/5)	USA	The BRAINS program
29	(Chen et al., 2020)	Supernormal	24 (16/8)	USA	The ADNI
30	(Kim et al., 2020)	Superager	35 (29/6)	South Korea	Community dwellers
31	(Borelli et al., 2021)	Superager	10 (3/7)	Brazil	Community dwellers
32	(Gardener et al., 2021)	Superior cognitive performance	76 (44/32)	Australia	The AIBL study
33	(Gefen et al., 2021)	Superager	7 (7/0)	USA	The Northwestern's AD Center
34	(Park et al., 2021)	SCA	14 (7/7)	South Korea	The KFACS
35	(Wang & Zhang, 2021)	Supernormal	40 (NA)	China	The ADNI
36	(de Godoy et al., 2021)	Superager	12 (NA)	UK	Community dwellers
37	(Katsumi et al., 2021)	Superager	17 (12/5)	USA	The BRAINS program
38	(Dominguez et al., 2021)	Top cognitive performance	140 (94/46)	USA	The NACC, and the 90+ Study
39	(J. Park et al., 2022)	SCA	14 (7/7)	South Korea	The KFACS
40	(Jia et al., 2022)	Superager	34 (22/12)	China	Memory clinic of China-Japan Friendship Hospital
41	(Katsumi et al., 2022)	Superager	19 (14/5)	USA	The SAGES study
42	(de Souza et al., 2022)	Superager	10 (7/3)	Brazil	Community dwellers
43	(Chen et al., 2022)	Successful ager	97 (72/25)	USA	The DLBS

44	(Spencer et al., 2022)	Superager	37 (27/10)	USA	The NSAP
45	(Linuma et al., 2022)	High cognitive function	22 (16/6)	Japan	Community dwellers
46	(C.-h. Park et al., 2022)	Superager	32 (26/6)	South Korea	Community dwellers
47	(Nassif et al., 2022)	Superager	6 (5/1)	USA	The Northwestern's AD Center
48	(Yang et al., 2022)	SCA	64 (39/25)	China	The BABRI
49	(Biswas et al., 2023)	Superior global cognitive performer	71 (44/27)	USA	The 90+ Study
50	(de Godoy et al., 2023)	Superager	14 (NA)	Brazil	Community dwellers
51	(Tobe et al., 2023)	High cognitive function	19 (13/6)	Japan	Community dwellers
52	(Garo-Pascual et al., 2023)	Superager	64 (38/26)	Spain	The Vallecas Project
53	(Klinedinst et al., 2023)	Superager	287 (NA)	USA	The UK Biobank
54	(Xu et al., 2023)	SCA	1060 (437/623)	China	The UK Biobank
55	(Pezzoli et al., 2023)	SCA	74 (50/24)	USA	The BACS
56	(Patel et al., 2024)	Exceptional episodic memory	556 (305/251)	USA	The LLFS
57	(Keenan et al., 2024)	Superager	20 (15/5)	Canada	The ADNI
58	(Garo-Pascual et al., 2024)	Superager	64 (38/26)	Spain	The Vallecas Project
59	(Harrison et al., 2024)	Maintainers	221 (133/88)	USA	The ADNI
60	(Diamond et al., 2024)	Superager	24 (16/8)	USA	The SuperAging Research Initiative database
61	(Dominguez et al., 2024)	Top cognitive performance	138 (85/53)	USA	The ADNI, and the 90+ Study
62	(Kim et al., 2024)	Superager	57 (45/12)	Korea	Community dwellers

Note. The number is ordered by the publication date and is consistent with other tables. **Abbreviations**: ABC, Aberdeen Birth Cohort; AD, Alzheimer's disease; ADGC, Alzheimer Disease Genetic Consortium; ADNI, Alzheimer's Disease Neuroimaging Initiative; AIBL, Australian Imaging, Biomarkers and Lifestyle; BABRI, Beijing Aging Brain Rejuvenation Initiative; BACS, Berkeley Aging Cohort Study; BRAINS, Brain Resilience in Aging: Integrated Neuroscience Studies; DLBS, Dallas Lifespan Brain Study; HABS, Harvard Aging Brain Study; Health ABC, Health, Aging, and Body Composition; JJP-VAMC, James J. Peters Veterans Affairs Medical Center; KFACS, Korean Frailty Aging Cohort Study; LCBC, Lifespan Changes in Brain and Cognition; LLFS, Long Life Family Study; MCSA, Mayo Clinic Study of Aging; NA, non-available; NACC, National Alzheimer's Coordinating Center; NIA-LOAD, National Institute

on Aging Late-Onset Alzheimer Disease; NSAP, Northwestern SuperAging Program; PACC, Preclinical Alzheimer Cognitive Composite; R/OCAS, Rochester/Orange County Aging Study; SAGES, Successful Aging after Elective Surgery; SCA, successful cognitive aging; SMADRC, Shiley-Marcos Alzheimer's Disease Research Center; UCSD, University of California, San Diego; UCSF, University of California San Francisco; UK, the United Kingdom; USA, the United State of America; WHICAP, Washington Heights Aging Project.

Table S3. Quality assessment of included studies.

		Risk of bias				Applicability		
**Num.**	**Study**	Patient Selection	Index Test	Reference Standard	Flow and Timing	Patient Selection	Index Test	Reference Standard
1	(Fjell et al., 2006)	Low	Low	Low	Low	Low	Low	Low
2	(Daffner et al., 2006)	Low	Unclear	Low	Low	Low	Low	Low
3	(Riis et al., 2008)	Unclear	Unclear	Low	Low	Low	Low	Low
4	(Waiter et al., 2008)	Unclear	Low	Low	Low	Low	Low	Low
5	(Rosano et al., 2012)	Low	Low	Low	Unclear	Low	Low	Low
6	(Silverman et al., 2012)	Low	Unclear	Low	Low	Low	Low	Low
7	(Harrison et al., 2012)	Unclear	Unclear	Low	Low	Low	Low	Low
8	(Josefsson et al., 2012)	Low	Low	Low	Unclear	Low	Low	Low
9	(Pudas et al., 2013)	Low	Low	Low	Unclear	Low	Low	Low
10	(Barral et al., 2014)	Unclear	Low	Low	Unclear	Low	Low	Low
11	(Gefen et al., 2015)	Unclear	High	Low	Unclear	Low	Low	Low
12	(Sun et al., 2016)	Low	Low	Low	Low	Low	Low	Low
13	(Lin, Ren, et al., 2017)	Low	Low	Low	Unclear	Low	Low	Low
14	(Bott et al., 2017)	Low	Unclear	Low	Unclear	Low	Low	Low
15	(Lin, Wang, et al., 2017)	Unclear	Low	Low	Unclear	Low	Low	Low
16	(Mapstone et al., 2017)	Low	Low	Low	Low	Low	Low	Low
17	(Dekhtyar et al., 2017)	Low	Low	Low	Unclear	Low	Low	Low
18	(Degerman et al., 2017)	Low	Low	Low	Unclear	Low	Low	Low
19	(Gefen et al., 2018)	Low	High	Low	Low	Low	Low	Low
20	(Janeczek et al., 2018)	Low	High	Low	Low	Low	Low	Low
21	(Huentelman et al., 2018)	Unclear	Unclear	Low	Unclear	Low	Low	Low
22	(Harrison et al., 2018)	Low	Low	Low	Unclear	Low	Low	Low
23	(Baran et al., 2018)	Unclear	Low	Low	Unclear	Low	Low	Low

24	(Wang et al., 2019)	Unclear	Low	Low	Unclear	Low	Low	Low
25	(Arenaza-Urquijo et al., 2019)	Unclear	Low	Low	Unclear	Low	Low	Low
26	(Dang, Harrington, et al., 2019)	Low	Low	Low	Unclear	Low	Low	Low
27	(Dang, Yassi, et al., 2019)	Low	Unclear	Low	Unclear	Low	Low	Low
28	(Zhang et al., 2020)	Low	Low	Low	Low	Low	Low	Low
29	(Chen et al., 2020)	Unclear	Low	Low	Unclear	Low	Low	Low
30	(Kim et al., 2020)	Low	Unclear	Low	Unclear	Low	Low	Low
31	(Borelli et al., 2021)	Unclear	Unclear	Low	Unclear	Low	Low	Low
32	(Gardener et al., 2021)	Low	Unclear	Low	Unclear	Low	Low	Low
33	(Gefen et al., 2021)	Low	High	Low	Low	Low	Low	Low
34	(Park et al., 2021)	Unclear	Low	Low	Unclear	Low	Low	Low
35	(Wang & Zhang, 2021)	Unclear	Low	Low	Unclear	Low	Low	Low
36	(de Godoy et al., 2021)	Low	Unclear	Low	Unclear	Low	Low	Low
37	(Katsumi et al., 2021)	Low	Low	Low	Low	Low	Low	Low
38	(Dominguez et al., 2021)	Unclear	Low	Low	Unclear	Low	Low	Low
39	(J. Park et al., 2022)	Unclear	Low	Low	Unclear	Low	Low	Low
40	(Jia et al., 2022)	High	Unclear	Low	Unclear	Low	Low	Low
41	(Katsumi et al., 2022)	Low	Unclear	Low	Unclear	Low	Low	Low
42	(de Souza et al., 2022)	Unclear	Low	Low	Unclear	Low	Low	Low
43	(Chen et al., 2022)	Low	Low	Low	Unclear	Low	Low	Low
44	(Spencer et al., 2022)	Unclear	Low	Low	Unclear	Low	Low	Low
45	(Linuma et al., 2022)	Low	Low	Low	Unclear	Low	Low	Low
46	(C.-h. Park et al., 2022)	Low	Unclear	Low	Unclear	Low	Low	Low
47	(Nassif et al., 2022)	Low	High	Low	Low	Low	Low	Low
48	(Yang et al., 2022)	Low	Low	Low	Low	Low	Low	Low
49	(Biswas et al., 2023)	Low	High	Low	Low	Low	Low	Low
50	(de Godoy et al., 2023)	Low	High	Low	Unclear	Low	Low	Low

51	(Tobe et al., 2023)	Low	Low	Low	Unclear	Low	Low	Low
52	(Garo-Pascual et al., 2023)	Low	Unclear	Low	Unclear	Low	Low	Low
53	(Klinedinst et al., 2023)	Low	Low	Low	Unclear	Low	Low	Low
54	(Xu et al., 2023)	Unclear	Low	Low	Unclear	Low	Low	Low
55	(Pezzoli et al., 2023)	Unclear	Low	Low	Unclear	Low	Low	Low
56	(Patel et al., 2024)	Low	Low	Low	Unclear	Low	Low	Low
57	(Keenan et al., 2024)	Unclear	Low	Low	Unclear	Low	Low	Low
58	(Garo-Pascual et al., 2024)	Unclear	Low	Low	Unclear	Low	Low	Low
59	(Harrison et al., 2024)	Low	Low	Low	Unclear	Low	Low	Low
60	(Diamond et al., 2024)	Low	Unclear	Low	Unclear	Low	Low	Low
61	(Dominguez et al., 2024)	Low	Low	Low	Unclear	Low	Low	Low
62	(Kim et al., 2024)	Low	High	Low	Unclear	Low	Low	Low

Note. the number is ordered by the publication date and is consistent with other tables.

Table S4. Cognitive tests used for SCA definition in the included articles.

Cognitive tests	Times used	Article Number
TMT-B	31	1, 38, 61, 40, 48, 15, 23, 24, 29, 35, 59, 7, 11, 19, 20, 21, 31, 33, 36, 44, 47, 50, 60, 12, 28, 37, 22, 41, 54, 57, 55
Category fluency	26	2, 3, 15, 23, 24, 29, 35, 59, 7, 11, 19, 20, 21, 31, 33, 36, 44, 47, 50, 60, 26, 27, 32, 52, 58, 55
Rey AVLT	25	16, 40, 42, 48, 13, 15, 23, 24, 29, 35, 59, 7, 11, 19, 20, 21, 31, 33, 36, 44, 47, 50, 60, 57, 62
BNT	20	2, 3, 40, 7, 11, 19, 20, 21, 31, 33, 36, 44, 47, 50, 60, 32, 52, 58, 55, 62
Digit span	16	1, 2, 3, 34, 39, 15, 23, 24, 29, 35, 59, 26, 27, 32, 55, 62
Logical memory (WMS)	15	2, 3, 10, 56, 38, 61, 13, 15, 23, 24, 29, 35, 59, 32, 55
SDMT	15	48, 15, 23, 24, 29, 35, 43, 59, 26, 27, 32, 52, 58, 54, 55
MMSE	11	6, 34, 39, 49, 13, 15, 23, 24, 29, 35, 59
CVLT	10	38, 61, 12, 28, 37, 22, 26, 27, 32, 55
TMT-A	10	1, 40, 48, 15, 23, 24, 29, 35, 59, 55
CDT	9	45, 51, 48, 15, 23, 24, 29, 35, 59
ADAS-cog	7	13, 15, 23, 24, 29, 35, 59
Stroop test	7	1, 48, 26, 27, 32, 55, 62
COWAT	5	1, 2, 3, 32, 62
ROCF	4	48, 30, 46, 62
Selective reminding test	4	10, 56, 52, 58
Benton visual retention test	2	10, 56
Block design (WASI)	2	1, 40
CCRT	2	45, 51
CPMT	2	45, 51
Frontal assessment battery screening tool	2	34, 39
RPM	2	4, 43
Similar word task	2	45, 51
SVLT	2	30, 46

Visual retention test	2	2, 3
Word list test	2	34, 39
Word recall task	2	45, 51
HVLT	2	41, 43
3MS test	1	5
CANTAB DMS	1	43
CANTAB SRM	1	43
CANTAB SWM	1	43
CANTAB SOC	1	43
CANTAB VRM	1	43
CBTT	1	1
CDR	1	6
Digit Comparison	1	43
ETS Letter Sets	1	43
FIT	1	53
FNAME	1	17
LNS	1	43
Matrix reasoning (WASI)	1	1
MCT	1	17
MHT No.12	1	4
List Sorting Task	1	43
Operation Span	1	43
PACC	1	59
Pair-matching test	1	54
Pattern Comparison	1	43
Rey CFT	1	32
SRT	1	17
Visual reproduction	1	55

Woodcock-Johnson III Memory for Names immediate and delayed recognition	1	43

Note. the number is ordered by the publication date and is consistent with other tables. **Abbreviations:** 3MS, Modified Mini Mental State test; ADAS-cog, Alzheimer's disease assessment scale-cognitive section; AVLT: Auditory Verbal Learning Test; BNT, Boston Naming Test; CANTAB, Cambridge Neuropsychological Test Automated Battery; CBTT, Corsi Block Tapping Test; CCRT, Category Cued Recall Task; CDR: Clinical Dementia Rating; CDT, Clock Drawing Task; CFT, complex figure test; COWAT, Controlled Oral Word Association Test; CPMT, Character Position Matching Task; CVLT, California Verbal Learning Test; DMS, Delayed Match to Sample; ETS, Educational Testing Service; FNAME, Face Name Associative Memory Exam; HVLT, Hopkins Verbal Learning Test; LNS, Letter Number Sequencing; MCT, Memory Capacity Test; MHT, Moray House Test; MMSE: Mini-Mental State Examination; PACC, Preclinical Alzheimer Cognitive Composite; ROCF, Rey-Osterrieth Complex Figure test; RPM, Raven's Standard Progressive Matrices test; SDMT, Symbol Digit Modalities Test; SOC, Stockings of Cambridge; SRM, Spatial Recognition Memory; SRT, Selective Reminding Test; SVLT, Seoul Verbal Learning Test; SWM, Spatial Working Memory; TMT-A, Trail-Making Test Part A; TMT-B, Trail-Making Test Part B; VRM, Verbal Recognition Memory; WASI, Wechsler Abbreviated Scale of Intelligence; WMS, Wechsler Memory Scale.

Table S5. Genetic and epigenetic biomarkers of successful cognitive aging.

Num.	Publication	Definition	Demographics	Biomarkers	Main results
8	(Josefsson et al., 2012)	LTD	285 (18%) were classified as maintainers, 209 (13%) as decliners, and 1,064 (68%) as the average group.	*APOE*, *KIBRA*, *BDNF*, *COMT*	*COMT*-met was a significant predictor of maintainers, and the *APOE* e4 allele was more frequent in decliners.
10	(Barral et al., 2014)	CCD	18 families from the LLFS, including 467 participants.	Genome-wide linkage analysis	The exceptional EM was linked to the 6q24 region, including SNP rs6902875, especially among non-*APOE* ε4 carriers.
11	(Gefen et al., 2015)	GCD	Superagers: n=31 (F: 68%), age 82.52±2.93; middle-aged controls: n=18 (F: 67%), age 58.39±3.70; elderly controls: n=21 (F: 38%), age 83.76±4.0.	*APOE*	The frequency of ε4 alleles across superager and elderly control groups did not differ statistically.
14	(Bott et al., 2017)	GCD	Resilient-agers: n=17 (F: 42%), age 69.2±0.96; Average-agers: n=56 (F: 51%), age 70.9±0.65; Sub-agers: n=47 (F: 47%), age 72.0±0.68.	*APOE*, *CR1*	Resilient-agers displayed a higher frequency of *APOE* e4 and *CR1* AA/AG alleles than sub-agers but not average agers.
15	(Lin, Wang, et al., 2017)	LTD	Declining agers, n=75; Successful agers, n=144; Low stable agers, n=135	*APOE*	*APOE* e4 carriers were more likely to be in low stable agers compared to successful agers.
17	(Dekhtyar et al., 2017)	CCD	Optimal performers: n=25 (F: 16), age 77.5±6.75; Typical performers: n=100 (F: 53), age 78.89±5.5.	*APOE*	Optimal or typical performers had no differences in *APOE* e4 carrying rate.
18	(Degerman et al., 2017)	LTD	The maintainers: n=16 (F:8), age 57.8±3.6; the decliners: n=16 (F:8), age 57.9±3.6; and the averages: n=20 (F: 9), age 58.0±3.5.	DNA methylation	A lower delta DNAm age was observed for the maintainers compared with average or accelerated decliners, and the DNAm age was a significant predictor of dementia.
21	(Huentelman et al., 2018)	GCD	Superagers: n=56 (F:39), age 83.0±3.3; Cognitively-average controls: n=22 (F:3), age 82.8±2.6.	WES; *MAP2K3*	Superaging was associated with variants in the *MAP2K3* gene. Three SNPs contributed to the

					significance (rs2363221 [intron 1], rs2230435 [exon 5], rs736103 [intron 7]).
22	(Harrison et al., 2018)	GCD	Successful agers (SA): n=26 (F: 23), age 74.9±4.6; TOAs: n=103 (F: 55), age 75.9±4.5.	*APOE*	There was no difference in *APOE* ε4 carriage rate between SA and TOA.
23	(Baran et al., 2018)	LTD	AD: n=27 (F:14), age 73.18±7.34; MCI: n=69 (F:34), age 71.27±7.84; NC: n=172 (F:70), age 74.56±6.17; and supernormals (SN): n=122 (F:72), age 73.88±6.64.	*APOE*	The SN group had fewer *APOE4+* carriers than the AD group and MCI group, but no significant difference between the SN and NC groups.
26	(Dang, Harrington, et al., 2019)	GCD	Superagers: n = 179 (F: 53.6%), mean age 68.43; CNFA: n = 179 (F: 53.6%), mean age 68.53.	*APOE*	The prevalence of *APOE* ε4 was equivalent between Superagers and CNFA.
27	(Dang, Yassi, et al., 2019)	GCD	Superagers: n = 172 (F: 55.81%), mean age 71.26; CNFA: n = 172 (F: 55.82%), mean age 72.25.	*APOE*	The baseline prevalence of *APOE* ε4 carriage was nearly equal between SA and CNFA.
29	(Chen et al., 2020)	LTD	Supernormals: n=24 (F: 16), age 72.56±5.54; average-ager controls: n=24 (F: 15), mean age 72.71±3.23.	*APOE*	No significant difference was found in *APOE* ε4 carrying rate between supernormals and average-agers.
32	(Gardener et al., 2021)	GCD	SCP: n=76 (F: 44), age 75.58±3.9; TOAs: n=100 (F: 56), age 76.70±4.4.	*APOE*	*APOE* e*4* showed no difference between the two groups.
34	(Park et al., 2021)	CCD	SCA group: n=14 (F: 7), age 76.4±3.6; NCA group: n=15 (F: 6), age 75.1±4.9.	DNA methylation	The SCA group showed significantly delayed intrinsic and extrinsic EAA than the NCA group.
39	(J. Park et al., 2022)	CCD	SCA group: n=14 (F:7), age 76.4±3.6; NCA group: n=15 (F: 6), age 75.1±4.9.	DNA methylation	*CEND*1 and *miR*885 were validated as having significantly different gene expressions between the SCA and NCA groups

44	(Spencer et al., 2022)	GCD	Superagers: n=37 (F: 27), mean age 82.7±2.8; controls: n=35 (F: 19), mean age 83.7±4.3.	The AD PHS	There was no significant difference in the AD PHS between Superagers and cognitively normal controls.
52	(Garo-Pascual et al., 2023)	GCD	Superagers: n=64 (F: 38), mean age 81.6; TOAs: n=55 (F: 35), mean age 82.1.	*APOE*	No between-group difference in *APOE* gene allelic composition was found.
55	(Pezzoli et al., 2023)	Multiple	Superagers (SA): (1) SA-ALL, n=74, (2) SA-CAG, n = 37, (3) SA-EM, n = 37, (4) SA-NM, n = 37, and (5) SA-CVLT, n = 31; and typical agers: n = 110.	*APOE*	No differences in *APOE* genotype were found across superager definitions compared with typical agers.
56	(Patel et al., 2024)	CCD	556 exceptional memory subjects from 27 families, age 66.26±15.28, F: 305; 3777 non-exceptional memory subjects from 403 families, age 71.72±15.93, F: 2081.	*APOE*	There were no significant differences in the proportion of distribution of the *APOE* e4 allele or *APOE* e2 allele between exceptional and non-exceptional families.

Abbreviations: AD, Alzheimer's disease; *APOE*, apolipoprotein E; *BDNF*, brain-derived neurotrophic factor; CAG, cognitive age gap; *CEND*1, cycle exit and neuronal differentiation 1; CI, confidence interval; CNFA, cognitively normal for age; *COMT*, catechol-O-methyltransferase; *CR1*, complement receptor 1; CVLT, California Verbal Learning Test; DNA, deoxyribonucleic acid; F: female; EAA, epigenetic age acceleration; EM, episodic memory; *KIBRA*, kidney and brain expressed protein; LLFS, Long-Life Family Study; *MAP2K3*, Mitogen-Activated Protein Kinase Kinase 3; *miR*885, microRNA 885; NC, normal cognition; NCA, normal cognitive aging; NM, non-memory cognition; PHS, polygenic hazard score; SA/SCA, successful cognitive aging; MADRC, Shiley Marcos Alzheimer's Disease Research Center; SCP, Older adults with superior cognitive performance; SNP, single-nucleotide polymorphisms; TOA, typical older adult; WES, Whole Exome Sequencing.

Table S6. Biofluid biomarkers of successful cognitive aging.

Num.	Publication	Definition	Demographics	Biomarkers	Main results
6	(Silverman et al., 2012)	CCD	Primary sample: 277 male veteran probands, aged 75+; replication sample: 202 relatives.	Blood CRP	Higher CRP in cognitively intact probands was associated with a lower risk of dementia in relatives.
14	(Bott et al., 2017)	GCD	Resilient-agers: n=17 (F: 42%), age 69.2±0.96; Average-agers: n=56 (F: 51%), age 70.9±0.65; Sub-agers: n=47 (F: 47%), age 72.0±0.68.	IL 6, LDL, HDL, and fasting insulin	Resilient-agers had lower levels of IL-6 and insulin than sub-agers but not average-agers.
15	(Lin, Wang, et al., 2017)	LTD	Declining agers, n=75; Successful agers, n=144; Low stable agers, n=135	CSF Aβ1-42 and t-tau	$A\beta_{1\text{-}42}$+ were more likely to be in low stable agers compared to successful agers. Individuals with t-tau+ were more likely to appear in declining agers compared to successful agers.
16	(Mapstone et al., 2017)	CCD	Supernormal: n= 41 (F:21), age 83.22±3.37; normal controls: n=41 (F: 21), age 83.29±3.82; and aMCI/AD: n=74 (F: 54), age 81.93±4.37.	Plasma metabolism	Significant differential abundance of 12 metabolites in those with superior memory relative to controls.
29	(Chen et al., 2020)	LTD	Supernormals: n=24 (F:16), age 72.56±5.54; average agers: n=24 (F: 15), age 72.71±3.23.	CSF Aβ/p-tau ratio	No significant difference was found in the CSF Aβ/p-tau ratio between supernormals and average agers.
52	(Garo-Pascual et al., 2023)	GCD	Superagers: n=64 (F: 38), mean age 81.6; TOAs: n=55 (F: 35), mean age 82.1.	$A\beta_{42}/A\beta_{40}$; total tau, p tau 181, p-tau 181/ $A\beta_{42}$, GFAP, NEFL.	No between-group difference in blood biomarkers was found.

56	(Patel et al., 2024)	CCD	556 exceptional memory subjects from 27 families, age 66.26±15.28, F: 305; 3777 non-exceptional memory subjects from 403 families, age 71.72±15.93, F: 2081.	WBC count, platelet count, high sensitivity CRP, and IL-6.	Participants from exceptional memory families had a higher monocyte count at baseline, and there was no significant difference in biomarker change over time.
62	(Kim et al., 2024)	GCD	Superager: n=57 (F:45), age 73.26±5.58; TOA: n=45 (F: 34), age 72.47±6.47.	Gut microbiome	The predictive model achieved acceptable AUC (0.861) and significant microbiome features for distinguishing superagers including Alistipes, PAC001137_g, PAC001138_g, Leuconostoc, and PAC001115_g.

Abbreviations: Aβ, Amyloid-β; AD, Alzheimer's disease; ADNI, Alzheimer's Disease Neuroimaging Initiative; aMCI, amnestic mild cognitive impairment; AUC, area under the curve; CI, confidence interval; CRP, C-reactive protein; CSF, cerebrospinal fluid; DMG, differentially methylated gene; EM, exceptional memory; F: female; GFAP, Glial fibrillary acidic protein; HDL, high-density lipoprotein; HR, hazard ratio; IL, Interleukin; LDL, low-density lipoprotein; LLFS, Long Life Family Study; NEFL, neurofilament light polypeptide; OR, odds ratio; p-tau, phosphorylated tau; t-tau, total tau; TOA, typical older adult; WBC, white blood cell.

Table S7. Histological biomarkers of successful cognitive aging.

Num.	Publication	Definition	Demographics	Biomarkers	Main results
11	(Gefen et al., 2015)	GCD	5 superagers: age 81-95, F: 5; 5 cognitively average agers: age 72-95, F: 4; and 5 aMCI patients: age 89-99, F: 3.	NFT, AP, and VEN density	Superagers showed a lower frequency of AD-type NFTs and AP, and a higher density of VENs than controls, particularly in the cingulate regions.
19	(Gefen et al., 2018)	GCD	5 younger controls (age 26-61), 5 superagers (age 81-95), 5 cognitively average elderly controls (age 72-95), 5 aMCI patients (age 89-99), and 5 AD patients (age 72-87)	Total neuronal density and VEN density	Superagers showed the highest mean VEN density in the anterior cingulate cortex, even when compared to younger cases.
20	(Janeczek et al., 2018)	GCD	2 children (age 2.5-10); 2 adolescents (age 13-19); 5 young adults (age 22-45); 3 middle-aged adults (age 50-57); 15 normal old adults (age 72-96); 5 superagers (age 87-95)	AChE-positive cortical pyramidal neurons	Superagers showed significantly lower staining intensity and density of AChE-positive cortical pyramidal neurons when compared with same-age peers.
33	(Gefen et al., 2021)	GCD	7 superagers: age 81-99, F: 7; 6 age-matched cognitively average normal control individuals: age 77-96, F: 4.	NFT and AP in the ERC.	Normal controls had Significantly more NFTs in ERC compared with superagers by a difference of about 3-fold. There were no significant differences in AP density.
47	(Nassif et al., 2022)	GCD	6 superagers (82-99 years old), 7 normal elderly (82-96 years old), 5 aMCI (89-99 years old), and 6 younger controls (26-61 years old).	Neuronal density and NFT	Superagers had larger soma size of layer II ERC neurons compared with all groups, and had fewer AD-related NFTs in layer II ERC than normal elderly.
49	(Biswas et al., 2023)	CCD	Superior global cognitive performers (SGCP): n=71 (F: 44), age 97.4±3.4; non-SGCP: n=31 (F: 17), mean age 98.0±2.9.	ADNC, CAA, Microinfarcts, HS, Atherosclerosis, Arteriolosclerosis, LBD, TDP-43.	ADNC and low levels of vascular pathologic change were not associated with superior cognition. Participants with limbic and neocortical LBD, or with HS were more likely to be non-SGCP. A high total burden of

	neuropathologic features is negatively associated with being SGCP.

Abbreviations: AChE, acetylcholinesterase; ADNC, Alzheimer's disease neuropathological change; AP, amyloid plaques; CAA, Cerebral amyloid angiopathy; ERC, entorhinal cortex; F, female; HS, Hippocampal sclerosis; NFT, neurofibrillary tangles; LBD, Lewy body disease; NSAP, Northwestern SuperAging Program; OR, odds ratio; TDP-43, TAR DNA-binding protein 43; VEN, von Economo neuron.

Table S8. PET biomarkers of successful cognitive aging.

Num.	Publication	Definition	Demographics	PET imaging type	Main results
13	(Lin, Ren, et al., 2017)	LTD	Supernormal: n=9 (F:8), age 73.53±6.38; healthy control: n=9 (F:8), age 72.31±5.57; MCI: n=9 (F:8), age 72.97±6.91.	FBP-PET	Supernormals had less Aβ deposition than MCI but showed no difference compared with healthy controls.
17	(Dekhtyar et al., 2017)	CCD	Optimal memory performers (OMP): n=25 (F:16), age 77.5±6.75; Typical memory performers (TMP): n=100 (F:53), age 78.89±5.5.	PiB-PET	OMP had no differences in Aβ burden compared with TMP. Non-maintainers showed higher Aβ burden at baseline in contrast with maintainers but didn't accumulate Aβ at a faster rate longitudinally.
22	(Harrison et al., 2018)	GCD	Successful agers (SA): n=26 (F:23), age 74.9±4.6; TOAs: n=103 (F:55), age 75.9±4.5.	PiB-PET	No difference in global PiB DVR exists between SA and TOA. Older SA individuals were less likely to have high brain Aβ.
23	(Baran et al., 2018)	LTD	AD: n=27 (F:14), age 73.18±7.34; MCI: n=69 (F:34), age 71.27±7.84; normal cognition (NC): n=172 (F:70), age 74.56±6.17; and supernormals (SN): n=122 (F:72), age 73.88±6.64.	FBP- and FDG-PET	SN had higher glucose metabolism than all others. Aβ burden and glucose metabolism in the right isthmus CC differed in SN compared to others, while SN glucose metabolism also differed from others in several frontal and temporal regions.
25	(Arenaza-Urquijo et al., 2019)	LTD	Full sample: n=475, age 83.5±3.21; including cognitive stable 80+, n= 192, age 82.7±2.8.	PiB- and FDG-PET	FDG-PET uptake in the ACC and anterior temporal pole was associated with baseline cognition in cognitively stable 80+, and predicted longitudinal cognitive change independent of Aβ status.
26	(Dang, Harrington, et al., 2019)	GCD	Superagers: n=179 (F: 53.6%), age 68.43; cognitively normal for age (CNFA): n=179 (F: 53.6%), age 68.53.	PiB-PET	The prevalence of Aβ+ was equivalent between Superagers and CNFA. In the absence of Aβ+, equivalent age-related changes in cognition were observed between Superagers and CNFA.

27	(Dang, Yassi, et al., 2019)	GCD	Superagers: n=172 (F: 55.81%), mean age 71.26; cognitively normal for age (CNFA): n=172 (F: 55.82%), mean age 72.25.	PiB-PET	The prevalence of Aβ carriage was nearly equal between SA and NA in baseline, and rates of age- and Aβ-associated atrophy did not differ between the groups on any measure.
31	(Borelli et al., 2021)	GCD	SA (n=10, F:3, age 82.1±2.5), C80 (older normal control, n=10, F:4, age 84.2±3.6) and C50 (younger normal control, n=10, F:2, age 58.5±5.8)	FDG- and PiB PET	SA group showed increased metabolic activity in the left and right subgenual ACC and bilateral hippocampus compared to the C80 group and showed similar metabolic activity with the C50 group. No significant Aβ differences between the SA and C80 groups.
32	(Gardener et al., 2021)	GCD	SCP: n=76 (F: 44), age 75.58±3.9; TOAs: n=100 (F: 56), age 76.70±4.4.	PiB-, FBP-, or FLUTE-PET	Aβ+ shows no difference between the two groups.
36	(de Godoy et al., 2021)	GCD	12 superagers and 13 age-matched controls	^{1}H-MRS	There was a higher total N-acetyl aspartate concentration in superagers than in age-matched controls using both approaches.
42	(de Souza et al., 2022)	CCD	Superagers: n=10 (F: 7), age 82.3±2.63; middle-aged controls (MC): n=10 (F: 9), age 58.70±5.52; age-matched controls (AC): n=10 (F: 7), age 83.5±4.22; AD: n=10 (F: 5), age 78.2±6.14.	PiB-PET	Superagers exhibited a similar Aβ load to AC and MC, differing in cognitive performance.
55	(Pezzoli et al., 2023)	Multiple	Superagers: (1) SA-ALL, n=74, (2) SA-CAG, n = 37, (3) SA-EM, n = 37, (4) SA-NM, n = 37, and (5) SA-CVLT, n = 31; and typical agers: n = 110.	FTP- and PiB-PET	No differences were found between SA groups and TA in global PiB DVR and the proportion of PiB-positive. Lower entorhinal FTP uptake was found in all SA groups.
59	(Harrison et al., 2024)	LTD	Maintainer: n=221 (F: 60%), age 74.1±6.2; Decliner: n=318 (F: 51%), age 77.1±5.9.	FBP or FBB PET and FTP-PET	Cognitive maintainers had less Aβ pathology in a cortical summary region, and lower tau pathology in

					both the ERC and the temporal Meta ROI; and there were no differences in rates of change.
61	(Dominguez et al., 2024)	CCD	The ADNI cohort: Amyloid PET sample (n=223, TCP: n=58, age 72.5±6.0, F: 34; non-TCP: n=165, age 73.0±6.2, F: 88); Tau PET sample (n=95, TCP: n=26, age 70.5±4.5, F: 18; non-TCP: n=69, age 69.4±5.1, F: 41). The 90+ study: Amyloid PET sample (n=171, TCP: n=41, age 91.9±1.5, F: 24; non-TCP: n=130, age 92.3±2.2, F: 80); Tau PET sample (n=49, TCP: n=13, age 91.8±1.4, F:9; non-TCP: n=36, age 91.5±1.3, F:17)	FBP- and FTP-PET	The results showed no group differences in amyloid SUVRs both regionally and in the whole cortex, and there were no differences in Braak composite SUVRs.

Abbreviations: Aβ, Amyloid β; ACC, anterior cingulate cortex; AD, Alzheimer's disease; ADNI, Alzheimer's Disease Neuroimaging Initiative; AIBL, Australian Imaging, Biomarkers, and Lifestyle; BACS, Berkeley Aging Cohort Study; CAG, cognitive age gap; CC, cingulate cortex; CVLT, California Verbal Learning Test; DVR, distribution volume ratio; ERC, entorhinal cortex; F, female; FBB, ^{18}F-florberaben; FBP, ^{18}F-Florbetapir; FDG, fluorodeoxyglucose; FLUTE, ^{18}F-Flutemetamol; FTP, ^{18}F-Flortaucipir; 1H-MRS, proton magnetic resonance spectroscopy; HABS, Harvard aging brain study; MCI, mild cognitive impairment; NM, non-memory cognition; PET, positron emission tomography; PiB, ^{11}C-Pittsburgh compound-B; ROI, region of interest; SCP, Older adults with superior cognitive performance; SUVR, TCP, top cognitive performance; TOA, typical older adult.

Table S9. Brain structural MRI biomarkers of successful cognitive aging.

Num.	Publication	Definition	Demographics	Neuroimaging biomarker type	Main results
1	(Fjell et al., 2006)	CCD	Young individuals: n=35, age 35.5±12.5; old individuals (including high/average fluid/executive function groups): n=39, age 70.7±7.0.	Cortical thickness	Large areas of cortex: old high fluid performers > average performers; the posterior cingulate and adjacent areas: old high fluid performers > young individuals.
5	(Rosano et al., 2012)	LTD	Cognitive maintainers: n=153 (F: 90), age 81.53±2.6; cognitive decliners: n=105 (F: 54), age 82.34±2.6.	WMH, GMV, MD, and FA	GMV of the medial temporal area: maintainers > decliners; MD of the cingulate cortex: maintainers < decliners.
7	(Harrison et al., 2012)	GCD	Superagers: n=12, age 83.5±3.0, elderly controls: n=10, age 83.1±3.4, and middle-aged controls: n=14, age 57.9±4.3.	Cortical thickness	Cerebral thickness: superager > elder controls; cerebral thickness of left ACC: superager > middle-aged controls; superagers displayed no atrophy compared to the middle-aged controls.
9	(Pudas et al., 2013)	LTD	Successful elders: n=51 (F: 38), age 68.8±7.1; average elders: n=51 (F: 23), age 68.8±6.9; young adults: n=45 (F: 22), age 35.3±7.1.	GMV and FA	Successful elders had smaller GMVs in the bilateral hippocampus and right parahippocampal gyrus than average elders, and there were no significant between-group differences in FA values.
11	(Gefen et al., 2015)	GCD	Superagers: n=31 (F: 68%), age 82.52±2.93; middle-aged controls: n=18 (F: 67%), age 58.39±3.70; elderly controls: n=21 (F: 38%), age 83.76±4.0.	Cortical thickness	Right ACC displayed greater thickness in Superagers compared with elderly controls and to the much middle-aged controls.
12	(Sun et al., 2016)	GCD	Young adults: n=41 (F: 51.2%), age 24.5±3.6; superagers: n=17 (F: 70.6%), age 67.8±6.0; and TOAs: n=23 (F: 34.8%), age 66.2±5.1.	Cortical thickness	Superagers have thicker brain regions in key paralimbic and limbic nodes of the default mode and salience networks.

14	(Bott et al., 2017)	GCD	Resilient-agers: n=17 (F: 42%), age 69.2±0.96; Average-agers: n=56 (F: 51%), age 70.9±0.65; Sub-agers: n=47 (F: 47%), age 72.0±0.68.	CC volumes	Resilient-agers had larger baseline cingulate cortex volumes than sub-agers but not average-agers.
17	(Dekhtyar et al., 2017)	CCD	Optimal memory performers (OMP): n=25 (F:16), age 77.5±6.75; Typical memory performers (TMP): n=100 (F:53), age 78.89±5.5.	Hippocampal volumes	OMPs had larger hippocampal volumes at baseline compared with TMPs, with no hippocampal volume changes between maintainers and non-maintainers (both OMPs at baseline) longitudinally.
22	(Harrison et al., 2018)	GCD	Successful agers (SA): n=26 (F:23), age 74.9±4.6; TOAs: n=103 (F:55), age 75.9±4.5.	Cortical thickness, WMH	SA had greater cortical thickness in the right ACC and prefrontal cortex and had greater hippocampal volume and lower WMH volumes. No differences in rates of brain atrophy were found.
29	(Chen et al., 2020)	LTD	Supernormals: n=24 (F: 16), age 72.56±5.54; average-ager controls: n=24 (F: 15), mean age 72.71±3.23.	White matter connectome	A unique structural connectome remains stable over time in supernormals relative to typical agers, and it significantly classifies positive vs. negative AD pathology at 72% accuracy.
30	(Kim et al., 2020)	GCD	Superager (SA): n=35 (F: 29), age 71.0±5.3; Typical ager (TA): n=55 (F: 46), age 73.0±5.6.	FA, MD, RD, and axial diffusivity	As compared to TA, SA demonstrated higher FA with lower MD, RD, and axial diffusivity in the corpus callosum and higher FA and lower RD in the right SLF.
32	(Gardener et al., 2021)	GCD	SCP: n=76 (F: 44), age 75.58±3.9; TOAs: n=100 (F: 56), age 76.70±4.4.	GMV and cortical thickness	SCPs had greater volume in the right rostral ACC, with no significant differences between rates of cortical thinning or volume atrophy between the two groups longitudinally.
35	(Wang & Zhang, 2021)	LTD	Supernormals: n=40; cognitively normal controls: n=45	White matter connectome	Older adults with stronger WM connections among right supramarginal, right pars opercularis, right pars triangularis, right rostral middle frontal, right insula,

					and right postcentral are more likely to be supernormals.
38	(Dominguez et al., 2021)	CCD	NACC: 70+ TCP (n=83), 70+ non-TCP (n=161), 80+ TCP (n=22), 80+ non-TCP (n=81); The 90+ study: 90+ TCP (n=35), 90+ non-TCP (n=73)	Cortical thickness	Thickness in cingulate regions can model TCP status with an AUC of 0.64, and a whole-brain, network-level approach outperformed the localist, cingulate models (AUC=0.74).
41	(Katsumi et al., 2022)	GCD	Superagers: n=19 (F: 74%), age 75.5±4.5; TOAs: n= 74 (F: 58%), age 75.9±4.0.	Cortical thickness	Superagers had greater baseline cortical thickness of the anterior mid-cingulate cortex, which predicted lower postoperative delirium severity scores in all patients.
48	(Yang et al., 2022)	CCD	SCA: n=64 (F:39), age 73.98 ± 3.70; MCI: n=68 (F:35), age: 75.84 ± 4.36, and cognitive normal controls (CNC): n=66 (F:32), age 74.64 ± 3.89.	GMV and GM network, WM connectome	GMV of the left MFG: SCA>CNC&MCI, and of the right hippocampus SCA>CNC>MCI; the left MFG covariant GM network is preserved for the SCA group; WM nodal efficiency differences: the frontal-basal ganglia regions (SCA>CNC&MCI), and the hippocampal related regions (SCA>CNC>MCI).
52	(Garo-Pascual et al., 2023)	GCD	Superagers: n=64 (F: 38), mean age 81.6; TOAs: n=55 (F: 35), mean age 82.1.	GMV	Superagers showed higher GMV in the MTL, cholinergic forebrain, and motor thalamus cross-sectionally, and showed slower total GM atrophy in the MTL, than did TOAs.
53	(Klinedinst et al., 2023)	LTD	Older potential superagers (n=287); Younger potential superagers (n=990); and other six groups (n=5844).	GMV	Superagers have the highest total GM volume and the best cognitive performance.
54	(Xu et al., 2023)	GCD	SCA group: n=1060 (F: 437), non-SCA group: n=6962.	GMV and cortical thickness, WM integrity	SCA had higher GMV in the left STG and the right Heschl’s gyrus and had higher mean thickness in the precentral and STG. An association was found

					between heightened cognitive aptitude and the integrity of WM structures within the brain.
55	(Pezzoli et al., 2023)	Multiple	Superagers (SA): (1) SA-ALL, n=74, (2) SA-CAG, n = 37, (3) SA-EM, n = 37, (4) SA-NM, n = 37, and (5) SA-CVLT, n = 31; and typical agers: n = 110.	Cortical thickness and hippocampal volume	Most SA groups showed greater cortical thickness compared to typical agers, especially in the anterior cingulate and midcingulate cortices and medial temporal lobes. Greater hippocampal volume was found in all SA groups except SA-NM.
58	(Garo-Pascual et al., 2024)	GCD	Superager: n=64 (F: 38), age 81.9±1.9; TOA: n=55 (F: 35), age 82.4±1.9.	White matter volume, integrity, and WMH	Superagers showed higher FA in frontal fibers and lower MD in most WM tracts cross-sectionally and showed slower FA decreases in all WM tracts assessed, slower MD increases in almost all WM tracts, all compared with TOAs.
59	(Harrison et al., 2024)	LTD	Maintainer: n=221 (F: 60%), age 74.1±6.2; Decliner: n=318 (F: 51%), age 77.1±5.9.	Hippocampal volumes	Maintainers had higher hippocampal volumes compared to decliners and had a lower rate of hippocampal atrophy compared to clinical converters.

Abbreviations: ACC, anterior cingulate cortex; AD, Alzheimer's disease; ADNI, Alzheimer's Disease Neuroimaging Initiative; AIBL, Australian Imaging Biomarkers, and Lifestyle; AUC, area under the curve; BACS, Berkeley Aging Cohort Study; CAG, cognitive age gap; CC, corpus callosum; CNC, cognitively normal control; CVLT, California Verbal Learning Test; DTI, diffusion-tensor imaging; DWI, diffusion-weighted imaging; EM, episodic memory; FA, fractional anisotropy; GM, gray matter; GMV, gray matter volume; HABS, Harvard Aging Brain Study; ITG, inferior temporal gyrus; MCI, mild cognitive impairment; MD, mean diffusivity; MFG, middle frontal gyrus; MTL, medial temporal lobe; NM, non-memory cognition; RD, radial diffusivity; SA, successful cognitive aging; SCP, Older adults with superior cognitive performance; SLF, superior longitudinal fasciculus; STG, superior temporal gyrus; TCP, top cognitive performance; TOA, typical older adult; WM, white matter; WMH, white matter hyperintensity.

Table S10. Preserved brain gray matter structures of SCA individuals revealed by studies shown in Table S9.

Region	Number of detections	Corresponding articles	Region (index) in HOA atlas
**Cingulate cortex**	**14**		
Anterior cingulate cortex	8	(Dominguez et al., 2021; Gardener et al., 2021; Gefen et al., 2015; Harrison et al., 2018; Harrison et al., 2012; Katsumi et al., 2022; Pezzoli et al., 2023; Sun et al., 2016)	Cingulate Gyrus, anterior division (290-291)
Posterior cingulate cortex	4	(Dominguez et al., 2021; Fjell et al., 2006; Gefen et al., 2015; Pezzoli et al., 2023)	Cingulate Gyrus, posterior division (300-301)
Cingulate gyrus isthmus	1	(Fjell et al., 2006)	Cingulate Gyrus, posterior division (300-301)
Paracingulate cortex	1	(Sun et al., 2016)	Paracingulate Gyrus (280-281)
**Medial temporal lobe**	**13**		
Hippocampus	7	(Dekhtyar et al., 2017; Garo-Pascual et al., 2023; Harrison et al., 2024; Harrison et al., 2018; Pezzoli et al., 2023; Rosano et al., 2012; Yang et al., 2022)	Hippocampus (6017, 6053)
Parahippocampal gyrus	3	(Garo-Pascual et al., 2023; Pezzoli et al., 2023; Rosano et al., 2012)	Parahippocampal Gyrus (340-341, 350-351)
Entorhinal cortex	3	(Garo-Pascual et al., 2023; Pezzoli et al., 2023; Rosano et al., 2012)	NA
**Frontal lobe**	**11**		
Medial prefrontal cortex	2	(Harrison et al., 2018; Sun et al., 2016)	Frontal Medial Cortex (250-251)
Medial orbitofrontal cortex	1	(Pezzoli et al., 2023)	Frontal Medial Cortex (250-251)
Dorsolateral prefrontal cortex	1	(Sun et al., 2016)	Middle Frontal Gyrus (40-41)
Fronto-marginal gyrus	1	(Fjell et al., 2006)	NA
Frontal operculum	1	(Sun et al., 2016)	Frontal Operculum Cortex (410-411)

Inferior frontal gyrus	1	(Sun et al., 2016)	Inferior Frontal Gyrus, pars triangularis (50-51)
Middle frontal gyrus	1	(Yang et al., 2022)	Middle Frontal Gyrus (40-41)
Superior frontal gyrus	1	(Sun et al., 2016)	Superior Frontal Gyrus (30-31)
Orbital gyrus	1	(Fjell et al., 2006)	Frontal Orbital Cortex (330-331)
Rectus gyrus	1	(Fjell et al., 2006)	NA
**Other subcortical structures**	**8**		
Insula	3	(Fjell et al., 2006; Harrison et al., 2018; Sun et al., 2016)	Insular Cortex (20-21)
Subcallosal gyrus	1	(Fjell et al., 2006)	Subcallosal Cortex (270-271)
Basal forebrain	1	(Garo-Pascual et al., 2023)	NA
Amygdala	1	(Garo-Pascual et al., 2023)	Amygdala (7018, 7054)
Thalamus	1	(Garo-Pascual et al., 2023)	Thalamus (2010, 2049)
**Temporal lobe**	**5**		
Inferior temporal gyrus	2	(Fjell et al., 2006; Pezzoli et al., 2023)	Inferior Temporal Gyrus, anterior division (140-141), and posterior division (150-151)
Middle temporal gyrus	2	(Fjell et al., 2006; Sun et al., 2016)	Middle Temporal Gyrus, anterior division (110-111) and posterior division (120-121)
Superior temporal gyrus	1	(Xu et al., 2023)	Superior Temporal Gyrus, anterior division (90-91)
**Parieto-occipital lobe**	**4**		
Angular gyrus	2	(Garo-Pascual et al., 2023; Sun et al., 2016)	Angular Gyrus (210-211)
Cuneus gyrus	1	(Fjell et al., 2006)	Cuneal Cortex (320-321)

Abbreviations: HOA, Harvard Oxford Atlas; NA, non-available.

Table S11. Preserved brain white matter structures of SCA individuals revealed by studies shown in Table S9.

Fibers	Integrity index	Corresponding articles
Anterior thalamic radiation	FA, MD	(Garo-Pascual et al., 2024)
Cingulum bundle	FA, MD	(Garo-Pascual et al., 2024; Rosano et al., 2012)
Corpus callosum	FA, MD, RD, and AD	(Kim et al., 2020)
External capsule	FA, MD	(Xu et al., 2023)
Forceps major	FA, MD	(Garo-Pascual et al., 2024)
Forceps minor	FA, MD	(Garo-Pascual et al., 2024; Kim et al., 2020)
Fornix crus + stria terminalis	FA, MD	(Xu et al., 2023)
Inferior fronto-occipital fasciculus	FA, MD	(Garo-Pascual et al., 2024)
Inferior longitudinal fasciculus	FA, MD, RD	(Garo-Pascual et al., 2024; Kim et al., 2020)
Superior longitudinal fasciculus	FA, MD, RD	(Garo-Pascual et al., 2024; Kim et al., 2020)

Abbreviations: FA, fractional anisotropy; MD, mean diffusivity; RD, radial diffusivity; AD, axial diffusivity.

Table S12. Brain functional neuroimaging biomarkers of successful cognitive aging.

Num.	Publication	Definition	Demographics	Neuroimaging type	Main results
2	(Daffner et al., 2006)	CCD	Cognitively high performers: old (n=16, age 73.0±4.9), middle-aged (n=16, age 50.3±3.4) and young (n=16, age 21.5±1.0) participants	ERP with a visual novelty oddball task	Across older but not younger subjects, cognitively high performers generate a larger anteriorly distributed P3 response to novel relative to standard stimuli.
3	(Riis et al., 2008)	CCD	Young adults: average-performer (AP, n=16, age 21.8±2.7), high-performer (HP, n=16, age 21.5±1.0); Middle-age adults: AP (n=15, age 49.2±3.1), HP (n=16, age 50.3±3.4); older adults: AP (n=15, age 70.1±4.3), HP (n=16, age 73.0±4.9).	ERP with a visual novelty oddball task	Cognitively high-performing old subjects allocated more attentional resources, as indexed by the novelty P3 response, than cognitively high-performing middle-aged and young subjects, and cognitively average-performing old subjects.
4	(Waiter et al., 2008)	CCD	Sustainer group: n=25 (F:11), age 69.8±0.6, decliner group: n=15 (F: 9), age 69.8±0.4	fMRI with an inspection time task	Sustainers showed more BOLD activation in the ACC region than the relative decliners, showed no significant difference compared with the young group, and the FC maps showed a similar pattern.
9	(Pudas et al., 2013)	LTD	Successful elders: n=51 (F: 38), age 68.8±7.1; average elders: n=51 (F: 23), age 68.8±6.9; young adults: n=45 (F: 22), age 35.3±7.1.	fMRI with an EM face-name paired-associate task	Successful elders had higher BOLD signals during encoding than average elders, notably in the bilateral PFC and the left hippocampus, whose activation was correlated with task performance.
13	(Lin, Ren, et al., 2017)	LTD	Supernormal: n=9 (F: 8), age 73.53±6.38; healthy control (HC): n=9 (F: 8), age 72.31±5.57; MCI: n=9 (F: 8), age 72.97±6.91.	rsfMRI: seed-based FC	Supernormals had significantly stronger FC between ACC and right hippocampus, middle CC (MCC) and left STG, and posterior CC (PCC) and right precuneus, while weaker FC between MCC and right MFG and MCC and right thalamus than other groups. All these FCs were significantly related to memory and global cognition.

24	(Wang et al., 2019)	LTD	data1 (Supernormals, SN: n=13, F: 8, age 76.46±7.52; average-ager controls, AC: n=16, F: 8, age 75.19±6.62); data 2 (SN+AC: n=18, F: 9, age 71.78±6.28; aMCI: n=57, F: 26, age 71.95±7.89; AD: n=26, F: 13, age 73.75±7.35)	rsfMRI: ALFF	A “Supernormal map” was identified, including the right FFG, right MFG, right ACC, left MTG, left PreCG, and left OFG. The map differentiated SN from AC and predicted a 1-year change in global cognition.
28	(Zhang et al., 2020)	GCD	Young adults: n=41 (F: 21), age 24.5±3.6; superagers: n=17 (F: 12), age 67.8±6.0; and TOAs: n=23 (F: 11), age 66.2±5.1	rsfMRI: FC	Within both DMN and SN, superagers had stronger FC compared with TOAs and similar FC compared with young adults, and stronger FC predicted better cognitive performance.
31	(Borelli et al., 2021)	GCD	SA (n=10, F:3, age 82.1±2.5), C80 (TOAs, n=10, F:4, age 84.2±3.6) and C50 (younger normal control, n=10, F:2, age 58.5±5.8)	rsfMRI: seed-based FC and ICA	The SA group showed decreased FC between the right subgenual ACC and PCC compared to the C80 group. ICA results showed the right SFG, a region within the IC–6 that distinguished SA from C80.
37	(Katsumi et al., 2021)	GCD	Young adults: n=41 (F: 51.2%), age 24.5±3.6; superagers: n=17 (F: 70.6%), age 67.8±6.0; and TOAs: n=23 (F: 34.8%), age 66.2±5.1	fMRI with a paired-associate recognition memory task	Superagers, like young adults, exhibited more distinct neural representations in the FFG and pHC while viewing visual stimuli belonging to different categories (greater neural differentiation) and more similar category representations between encoding and retrieval (greater neural reinstatement), compared with TOAs.
40	(Jia et al., 2022)	CCD	Superagers: n=34 (F: 22), age 68.47±6.51; TOAs: n=48 (F: 36), age 70.58±5.82.	rsfMRI: FC	The superagers had stronger cortical FC of Ch1-3 (nucleis of basal forebrain) with left putamen and insular cortex. The strength of FC positively correlated with cognition.
43	(Chen et al., 2022)	LTD	71 middle-aged adults (aged 35-54, superagers: 69%), 96 young-old adults (aged 55-69, superagers: 60%), 82 very old adults (aged 70-89, superagers:	fMRI with a subsequent memory paradigm	Successful agers exhibited high subsequent memory effect, at a level comparable to the young control group, until very old age; additional recruitment in prefrontal clusters, distant from the core task-related regions, were

			51%), and 41 young adults (aged 20-34).		identified in the left superior frontal and right orbitofrontal cortices in successful agers of young-old age.
45	(Linuma et al., 2022)	CCD	High cognitive function group: n=22 (F: 16), age 70±4.3; low cognitive function group: n=21 (F: 14), age 74.14±5.22.	EEG: temporal complexity	The high cognitive function group showed high complexity on a slower time scale in the frontal, parietal, and temporal lobes, reflecting the activation of long-distance neural interactions among various brain regions to achieve high cognitive functions.
46	(C.-h. Park et al., 2022)	GCD	Superagers: n=32 (F: 26), age 71.0±5.5; and typical agers: n=58 (F: 49), age 72.8±5.5.	rsfMRI: functional connectome	An ensemble learning method combining the three classifiers achieved the highest AUC (0.986). The most discriminative nodes for predicting superagers include the precuneus; posterior CC; insular cortex; and superior, middle, and inferior frontal gyrus.
50	(de Godoy et al., 2023)	GCD	Superagers: n=14; age 82.93±3.47; and TOAs: n=17; age 84.47±4.29.	rsfMRI: ICA	The default mode, salience, and language networks differentiated the two groups, and the most discriminative nodes were the precuneus, the PCC, the prefrontal cortex, the temporoparietal junction, the temporal pole, the extrastriate superior cortex, and the insula.
51	(Tobe et al., 2023)	CCD	High-cognitive function group: n=19 (F:13), age 69.05±3.03; Low-cognitive function group: n=19 (F: 13), age 72.63±3.25.	EEG: FC	Significant group differences with electrode dependence in the theta band, in particular, a high betweenness centrality was observed in the high-cognitive function group in the frontal region.
53	(Klinedinst et al., 2023)	LTD	Older potential superagers (n=287); Younger potential superagers (n=990); and other six groups (n=5844).	rsfMRI: ICA	Superagers had the lowest FC. The researchers suggest a novel hypothesis that super-agers possess enhanced neural processing efficiency that increases with age and introduce a definition of the “neural efficiency index.”

54	(Xu et al., 2023)	GCD	SCA group: n=1060 (F: 437), non-SCA group: n=6962.	rsfMRI: ICA	SCA group had enhanced resting-state network connectivity strength in the left FPN, the anterior DMN, and the basal ganglia network.
57	(Keenan et al., 2024)	GCD	Superagers: n=20 (F: 15), age 72.1±6.8; and Normal Ager: n=20 (F: 15) mean age 72.1±7.0.	rsfMRI: FC	Superagers generally do not demonstrate significantly stronger FC within the DMN or SN, and FC strength within these networks does not correlate with memory performance.
60	(Diamond et al., 2024)	GCD	Superagers: n=24 (F: 16), age 84.7±2.89; older-aged controls: n=16 (F:10), age 84.27±3.67.	rsfMRI: FC	Within-network and between-network FCs and segregation measurements of seven large-scale networks were not the primary differentiators between cognitively average aging and Superaging.

Abbreviations: ALFF, amplitude of low-frequency fluctuation; AUC, area under the curve; BOLD, blood oxygenation level dependent; CC, cingulate cortex; CEN, central executive network; DMN, default mode network; EEG, electroencephalography; ERP, event-related potential; FC, functional connectivity; FFG, fusiform gyrus; FI, fluid intelligence; fMRI, functional magnetic resonance imaging; FPN, frontoparietal network; MFG, middle frontal gyrus; ACC, anterior cingulate cortex; ICA, independent components analysis; MFG, middle frontal gyrus; MTG, middle temporal gyrus; OFG, orbitofrontal cortex; PCC, posterior cingulate cortex; PFC, prefrontal cortex; pHC, parahippocampal gyrus; PreCG, precentral gyrus; RF, random forest; rsfMRI, resting-state fMRI; SFG, superior frontal gyrus; SN, salience network; STG, superior temporal gyrus; TOA, typical older adult.

Table S13. Cross-domain relationships investigated in included studies that explored multidomain biomarkers.

Number	Publication	Interested Biomarkers	Main findings	Cross-domain relationships
1	(Pudas et al., 2013)	GMV	Successful older adults had smaller GMVs in the bilateral hippocampus and right parahippocampal gyrus than average older adults.	The functional differences were not driven by smaller GMVs in the average older adults.
		WM integrity	There were no significant between-group differences in FA values.	
		Task fMRI	Superagers had higher BOLD signals during encoding than average older adults, notably in the bilateral prefrontal cortex and the left hippocampus.	
2	(Gefen et al., 2015)	*APOE*4	SCA and NCA groups had no differences in APOE4 carrying rate.	No cross-domain relationship analysis.
		AP	Superagers showed a lower frequency of AP than controls.	
		NFT	Superagers showed a lower frequency of NFT than controls, especially in ERC, where controls had about 3-fold NFTs than superagers.	
		VEN	Superagers showed a higher density of VENs than controls, especially in the cingulate regions.	
		Cortical thickness	Superagers had greater cortical thickness in cingulate cortex than same-age controls and even had similar cortical thickness with young controls.	
3	(Lin, Ren, et al., 2017)	PET	Superagers showed no difference in Aβ deposition compared with controls.	No cross-domain relationship analysis.
		rsfMRI: FC	Superagers had significantly stronger or weaker FCs between the cingulate cortex and multiple regions, stronger FCs within DMN and SN, and stronger FCs of basal forebrain with putamen and insular cortex.	
4	(Bott et al., 2017)	*APOE*4	SCA and NCA groups had no differences in *APOE4* carrying rate.	No cross-domain relationship analysis.
		IL 6	No between-group difference was found in IL-6.	
5	(Lin, Wang, et al., 2017)	*APOE*4	*APOE4* carriers were more in low stable agers compared to successful agers.	No cross-domain relationship analysis.
		CSF Aβ	$A\beta_{1-42}$+ were more likely to be in low stable agers compared to successful agers.	
		CSF tau	t-tau+ were more likely to be in declining agers compared to successful agers.	

6	(Dekhtyar et al., 2017)	*APOE*4<br>PET Aβ<br>GMV	SCA and NCA groups had no differences in *APOE4* carrying rate.<br>Superagers showed no difference in Aβ deposition compared with controls.<br>Superagers had greater hippocampal GMV than controls.	No cross-domain relationship analysis.
7	(Harrison et al., 2018)	*APOE*4<br>PET Aβ<br>Cortical thickness<br>GMV<br>WMH	SCA and NCA groups had no differences in *APOE4* carrying rate.<br>Superagers showed no difference in Aβ deposition compared with controls.<br>Superagers had greater cortical thickness in multiple regions than same-age controls, especially in the cingulate cortex, prefrontal cortex, etc.<br>Superagers had greater hippocampal GMV than controls.<br>Superagers had lower WMH volumes than controls.	No cross-domain relationship analysis.
8	(Baran et al., 2018)	*APOE*4<br>PET Aβ<br>Glucose metabolism	SCA and NCA groups had no differences in *APOE4* carrying rate.<br>Superagers had less Aβ burden compared to controls in the cingulate cortex.<br>Superagers had higher glucose metabolism than controls in multiple regions, especially in the cingulate cortex.	No cross-domain relationship analysis.
9	(Dang, Harrington, et al., 2019)	*APOE*4<br>PET Aβ	SCA and NCA groups had no differences in *APOE4* carrying rate.<br>Superagers showed no difference in Aβ deposition compared with controls.	No cross-domain relationship analysis.
10	(Dang, Yassi, et al., 2019)	*APOE*4<br>PET Aβ	SCA and NCA groups had no differences in *APOE4* carrying rate.<br>Superagers showed no difference in Aβ deposition compared with controls.	No cross-domain relationship analysis.
11	(Chen et al., 2020)	*APOE*4<br>CSF Aβ<br>CSF tau<br>WM connectome	SCA and NCA groups had no differences in *APOE4* carrying rate.<br>No between-group difference was found in CSF Aβ.<br>No between-group difference was found in CSF tau.<br>Superagers had stronger WM connections and greater efficiency among multiple regions.	No cross-domain relationship analysis.
12	(Borelli et al., 2021)	PET Aβ<br>Glucose metabolism<br>rsfMRI: FC	Superagers showed no difference in Aβ deposition compared with controls.<br>Superagers had higher glucose metabolism than controls in multiple regions, especially in the cingulate cortex.<br>Superagers had significantly stronger or weaker FCs between the cingulate cortex and multiple regions.	No cross-domain relationship analysis.

		rsfMRI: ICA	Superagers had increased FC of the right superior frontal gyrus with the independent component 6 than controls.	
13	(Gardener et al., 2021)	*APOE*4<br>PET Aβ<br>GMV	SCA and NCA groups had no differences in *APOE4* carrying rate.<br>Superagers showed no difference in Aβ deposition compared with controls.<br>Superagers had greater GMV than controls in anterior cingulate, with no significant differences between rates of cortical thinning or volume atrophy between the two groups longitudinally.	Within only Aβ negative individuals, again there were no significant difference on rates of thinning or volume atrophy between two groups.
14	(Garo-Pascual et al., 2023)	*APOE*4<br>CSF Aβ<br>CSF tau<br>GMV	SCA and NCA groups had no differences in *APOE4* carrying rate.<br>No between-group difference was found in CSF Aβ.<br>No between-group difference was found in CSF tau.<br>Superagers had greater GMV than controls in multiple regions, including the medial temporal area, basal forebrain, thalamus, etc.	No cross-domain relationship analysis.
15	(Klinedinst et al., 2023)	GMV<br>rsfMRI: ICA	Superagers had the highest total brain GMV than other groups.<br>Superagers had the lowest FC and possessed enhanced neural processing efficiency.	No cross-domain relationship analysis.
16	(Xu et al., 2023)	Cortical thickness<br>GMV<br>WM integrity<br>rsfMRI: ICA	Superagers had greater cortical thickness than controls in multiple regions, including precentral, superior temporal region, etc.<br>Superagers had greater GMV than controls in multiple regions.<br>Superagers had higher FA and lower MD than controls in multiple fibers.<br>SCA group had enhanced resting-state network connectivity strength in the left FPN, the anterior DMN, and the basal ganglia network.	No cross-domain relationship analysis.
17	(Pezzoli et al., 2023)	*APOE*4<br>PET Aβ<br>PET tau<br>Cortical thickness	SCA and NCA groups had no differences in *APOE4* carrying rate.<br>Superagers showed no difference in Aβ deposition compared with controls.<br>Superager had lower tau deposition compared with controls, especially in ERC<br>Superagers had greater cortical thickness than controls in multiple regions especially in the cingulate cortex, and the medial temporal lobe, etc.	No cross-domain relationship analysis.

		GMV	Superagers had greater hippocampal GMV than controls.	
18	(Patel et al., 2024)	*APOE*4	SCA and NCA groups had no differences in *APOE4* carrying rate.	No cross-domain relationship analysis.
		APOE2	SCA and NCA groups had no differences in *APOE2* carrying rate.	
		CRP	No between-group difference was found in CRP.	
		WBC	Participants from SCA families had a higher monocyte count at baseline.	
		IL-6	No between-group difference was found in IL-6.	
19	(Harrison et al., 2024)	PET Aβ	Superagers had less global Aβ burden compared to decliners.	No cross-domain relationship analysis.
		PET tau	Superagers had lower tau deposition compared with decliners	
		GMV	Superagers had greater hippocampal GMV than decliners.	

Abbreviations:

Aβ, Amyloid-β; AP, amyloid plaques; *APOE*, apolipoprotein E allele; BOLD, blood oxygenation level dependent; CRP, C-reactive protein; CSF, cerebrospinal fluid; DMN, default mode network; ERC, entorhinal cortex; FA, fractional anisotropy; FC, functional connectivity; fMRI, functional magnetic resonance imaging; FPN, frontoparietal network; GMV, gray matter volume; ICA, independent components analysis; IL, Interleukin; MD, mean diffusivity; NCA, normal cognitive aging; NFT, neurofibrillary tangles; PET, positron emission tomography; SCA, successful cognitive aging; SN, salience network; VEN, von Economo neuron; WBC, white blood cell; WM, white matter; WMH, white matter hyperintensity.

References

Arenaza-Urquijo, E. M., Przybelski, S. A., Lesnick, T. L., Graff-Radford, J., Machulda, M. M., Knopman, D. S., Schwarz, C. G., Lowe, V. J., Mielke, M. M., Petersen, R. C., Jack, C. R., & Vemuri, P. (2019). The metabolic brain signature of cognitive resilience in the 80+: beyond Alzheimer pathologies. *Brain*, *142*(4), 1134-1147. https://doi.org/10.1093/brain/awz037

Baran, T. M., Lin, F. V., & Hanseeuw, B. (2018). Amyloid and FDG PET of Successful Cognitive Aging: Global and Cingulate-Specific Differences. *Journal of Alzheimer's Disease*, *66*(1), 307-318. https://doi.org/10.3233/jad-180360

Barral, S., Cosentino, S., Christensen, K., Newman, A. B., Perls, T. T., Province, M. A., & Mayeux, R. (2014). Common Genetic Variants on 6q24 Associated With Exceptional Episodic Memory Performance in the Elderly. *JAMA Neurology*, *71*(12). https://doi.org/10.1001/jamaneurol.2014.1663

Biswas, R., Kawas, C., Montine, T. J., Bukhari, S. A., Jiang, L., Corrada, M. M., & Abner, E. (2023). Superior Global Cognition in Oldest-Old Is Associated with Resistance to Neurodegenerative Pathologies: Results from The 90+ Study. *Journal of Alzheimer's Disease*, *93*(2), 561-575. https://doi.org/10.3233/jad-221062

Borelli, W. V., Leal-Conceição, E., Andrade, M. A., Esper, N. B., Feltes, P. K., Soder, R. B., Matushita, C. S., Hartmann, L. M., Radaelli, G., Schilling, L. P., Moriguchi-Jeckel, C., Marques da Silva, A. M., Portuguez, M. W., Franco, A. R., & da Costa, J. C. (2021). Increased Glucose Activity in Subgenual Anterior Cingulate and Hippocampus of High Performing Older Adults, Despite Amyloid Burden. *Journal of Alzheimer's Disease*, *81*(4), 1419-1428. https://doi.org/10.3233/jad-210063

Bott, N. T., Bettcher, B. M., Yokoyama, J. S., Frazier, D. T., Wynn, M., Karydas, A., Yaffe, K., & Kramer, J. H. (2017). Youthful Processing Speed in Older Adults: Genetic, Biological, and Behavioral Predictors of Cognitive Processing Speed Trajectories in Aging. *Frontiers in Aging Neuroscience*, *9*. https://doi.org/10.3389/fnagi.2017.00055

Chen, Q., Baran, T. M., Rooks, B., O'Banion, M. K., Mapstone, M., Zhang, Z., & Lin, F. (2020). Cognitively supernormal older adults maintain a unique structural connectome that is resistant to Alzheimer's pathology. *NeuroImage: Clinical*, *28*. https://doi.org/10.1016/j.nicl.2020.102413

Chen, X., Rundle, M. M., Kennedy, K. M., Moore, W., & Park, D. C. (2022). Functional activation features

of memory in successful agers across the adult lifespan. *NeuroImage*, *257*. https://doi.org/10.1016/j.neuroimage.2022.119276

Daffner, K. R., Ryan, K. K., Williams, D. M., Budson, A. E., Rentz, D. M., Wolk, D. A., & Holcomb, P. J. (2006). Age-related differences in attention to novelty among cognitively high performing adults. *Biological Psychology*, *72*(1), 67-77. https://doi.org/10.1016/j.biopsycho.2005.07.006

Dang, C., Harrington, K. D., Lim, Y. Y., Ames, D., Hassenstab, J., Laws, S. M., Yassi, N., Hickey, M., Rainey-Smith, S. R., Robertson, J., Rowe, C. C., Sohrabi, H. R., Salvado, O., Weinborn, M., Villemagne, V. L., Masters, C. L., & Maruff, P. (2019). Superior Memory Reduces 8-year Risk of Mild Cognitive Impairment and Dementia But Not Amyloid β-Associated Cognitive Decline in Older Adults. *Archives of Clinical Neuropsychology*, *34*(5), 585-598. https://doi.org/10.1093/arclin/acy078

Dang, C., Yassi, N., Harrington, K. D., Xia, Y., Lim, Y. Y., Ames, D., Laws, S. M., Hickey, M., Rainey-Smith, S., Sohrabi, H. R., Doecke, J. D., Fripp, J., Salvado, O., Snyder, P. J., Weinborn, M., Villemagne, V. L., Rowe, C. C., Masters, C. L., Maruff, P.,…Woodward, M. (2019). Rates of age- and amyloid β-associated cortical atrophy in older adults with superior memory performance. *Alzheimer's & Dementia: Diagnosis, Assessment & Disease Monitoring*, *11*(1), 566-575. https://doi.org/10.1016/j.dadm.2019.05.005

de Godoy, L. L., Studart-Neto, A., de Paula, D. R., Green, N., Halder, A., Arantes, P., Chaim, K. T., Moraes, N. C., Yassuda, M. S., Nitrini, R., Dresler, M., da Costa Leite, C., Panovska-Griffiths, J., Soddu, A., & Bisdas, S. (2023). Phenotyping Superagers Using Resting-State fMRI. *American Journal of Neuroradiology*, *44*(4), 424-433. https://doi.org/10.3174/ajnr.A7820

de Godoy, L. L., Studart-Neto, A., Wylezinska-Arridge, M., Tsunemi, M. H., Moraes, N. C., Yassuda, M. S., Coutinho, A. M., Buchpiguel, C. A., Nitrini, R., Bisdas, S., & da Costa Leite, C. (2021). The Brain Metabolic Signature in Superagers Using In Vivo 1H-MRS: A Pilot Study. *American Journal of Neuroradiology*. https://doi.org/10.3174/ajnr.A7262

de Souza, G. S., Andrade, M. A., Borelli, W. V., Schilling, L. P., Matushita, C. S., Portuguez, M. W., da Costa, J. C., & Marques da Silva, A. M. (2022). Amyloid-β PET Classification on Cognitive Aging Stages Using the Centiloid Scale. *Molecular Imaging and Biology*, *24*(3), 394-403.

https://doi.org/10.1007/s11307-021-01660-7

Degerman, S., Josefsson, M., Nordin Adolfsson, A., Wennstedt, S., Landfors, M., Haider, Z., Pudas, S., Hultdin, M., Nyberg, L., & Adolfsson, R. (2017). Maintained memory in aging is associated with young epigenetic age. *Neurobiology of Aging*, *55*, 167-171. https://doi.org/10.1016/j.neurobiolaging.2017.02.009

Dekhtyar, M., Papp, K. V., Buckley, R., Jacobs, H. I. L., Schultz, A. P., Johnson, K. A., Sperling, R. A., & Rentz, D. M. (2017). Neuroimaging markers associated with maintenance of optimal memory performance in late-life. *Neuropsychologia*, *100*, 164-170. https://doi.org/10.1016/j.neuropsychologia.2017.04.037

Diamond, B. R., Sridhar, J., Maier, J., Martersteck, A. C., & Rogalski, E. J. (2024). SuperAging functional connectomics from resting-state functional MRI. *Brain Communications*, *6*(4). https://doi.org/10.1093/braincomms/fcae205

Dominguez, E. N., Corrada, M. M., Kawas, C. H., & Stark, C. E. L. (2024). Resilience to AD pathology in Top Cognitive Performers. *Frontiers in Aging Neuroscience*, *16*. https://doi.org/10.3389/fnagi.2024.1428695

Dominguez, E. N., Stark, S. M., Ren, Y., Corrada, M. M., Kawas, C. H., & Stark, C. E. L. (2021). Regional Cortical Thickness Predicts Top Cognitive Performance in the Elderly. *Frontiers in Aging Neuroscience*, *13*. https://doi.org/10.3389/fnagi.2021.751375

Fjell, A. M., Walhovd, K. B., Reinvang, I., Lundervold, A., Salat, D., Quinn, B. T., Fischl, B., & Dale, A. M. (2006). Selective increase of cortical thickness in high-performing elderly—structural indices of optimal cognitive aging. *NeuroImage*, *29*(3), 984-994. https://doi.org/10.1016/j.neuroimage.2005.08.007

Gardener, S. L., Weinborn, M., Sohrabi, H. R., Doecke, J. D., Bourgeat, P., Rainey-Smith, S. R., Shen, K.-k., Fripp, J., Taddei, K., Maruff, P., Salvado, O., Savage, G., Ames, D., Masters, C. L., Rowe, C. C., Martins, R. N., & O'Bryant, S. (2021). Longitudinal Trajectories in Cortical Thickness and Volume Atrophy: Superior Cognitive Performance Does Not Protect Against Brain Atrophy in Older Adults. *Journal of Alzheimer's Disease*, *81*(3), 1039-1052. https://doi.org/10.3233/jad-201243

Garo-Pascual, M., Gaser, C., Zhang, L., Tohka, J., Medina, M., & Strange, B. A. (2023). Brain structure and

phenotypic profile of superagers compared with age-matched older adults: a longitudinal analysis from the Vallecas Project. *The Lancet Healthy Longevity*, *4*(8), e374-e385. https://doi.org/10.1016/s2666-7568(23)00079-x

Garo-Pascual, M., Zhang, L., Valent-Soler, M., & Strange, B. A. (2024). Superagers Resist Typical Age-Related White Matter Structural Changes. *The Journal of Neuroscience*, *44*(25). https://doi.org/10.1523/jneurosci.2059-23.2024

Gefen, T., Kawles, A., Makowski-Woidan, B., Engelmeyer, J., Ayala, I., Abbassian, P., Zhang, H., Weintraub, S., Flanagan, M. E., Mao, Q., Bigio, E. H., Rogalski, E., Mesulam, M. M., & Geula, C. (2021). Paucity of Entorhinal Cortex Pathology of the Alzheimer's Type in SuperAgers with Superior Memory Performance. *Cerebral Cortex*, *31*(7), 3177-3183. https://doi.org/10.1093/cercor/bhaa409

Gefen, T., Papastefan, S. T., Rezvanian, A., Bigio, E. H., Weintraub, S., Rogalski, E., Mesulam, M. M., & Geula, C. (2018). Von Economo neurons of the anterior cingulate across the lifespan and in Alzheimer's disease. *Cortex*, *99*, 69-77. https://doi.org/10.1016/j.cortex.2017.10.015

Gefen, T., Peterson, M., Papastefan, S. T., Martersteck, A., Whitney, K., Rademaker, A., Bigio, E. H., Weintraub, S., Rogalski, E., Mesulam, M. M., & Geula, C. (2015). Morphometric and Histologic Substrates of Cingulate Integrity in Elders with Exceptional Memory Capacity. *The Journal of Neuroscience*, *35*(4), 1781-1791. https://doi.org/10.1523/jneurosci.2998-14.2015

Harrison, T. M., Chadwick, T., Pezzoli, S., Lee, J., Landau, S. M., & Jagust, W. J. (2024). Cognitive Trajectories and Alzheimer Disease Biomarkers: From Successful Cognitive Aging to Clinical Impairment. *Annals of Neurology*, *96*(2), 378-389. https://doi.org/10.1002/ana.26964

Harrison, T. M., Maass, A., Baker, S. L., & Jagust, W. J. (2018). Brain morphology, cognition, and β-amyloid in older adults with superior memory performance. *Neurobiology of Aging*, *67*, 162-170. https://doi.org/10.1016/j.neurobiolaging.2018.03.024

Harrison, T. M., Weintraub, S., Mesulam, M. M., & Rogalski, E. (2012). Superior Memory and Higher Cortical Volumes in Unusually Successful Cognitive Aging. *Journal of the International Neuropsychological Society*, *18*(6), 1081-1085. https://doi.org/10.1017/s1355617712000847

Huentelman, M. J., Piras, I. S., Siniard, A. L., De Both, M. D., Richholt, R. F., Balak, C. D., Jamshidi, P., Bigio, E. H., Weintraub, S., Loyer, E. T., Mesulam, M. M., Geula, C., & Rogalski, E. J. (2018).

Associations of MAP2K3 Gene Variants With Superior Memory in SuperAgers. *Frontiers in Aging Neuroscience*, *10*. https://doi.org/10.3389/fnagi.2018.00155

Janeczek, M., Gefen, T., Samimi, M., Kim, G., Weintraub, S., Bigio, E., Rogalski, E., Mesulam, M. M., & Geula, C. (2018). Variations in Acetylcholinesterase Activity within Human Cortical Pyramidal Neurons Across Age and Cognitive Trajectories. *Cerebral Cortex*, *28*(4), 1329-1337. https://doi.org/10.1093/cercor/bhx047

Jia, S.-h., Zhou, Z., Shao, W., Zhou, X., Lv, S., Hong, W., & Peng, D.-t. (2022). The functional connectivity of basal forebrain is associated with superior memory performance in older adults: a case-control study. *BMC Geriatrics*, *22*(1). https://doi.org/10.1186/s12877-022-03226-w

Josefsson, M., de Luna, X., Pudas, S., Nilsson, L. G., & Nyberg, L. (2012). Genetic and Lifestyle Predictors of 15-Year Longitudinal Change in Episodic Memory. *Journal of the American Geriatrics Society*, *60*(12), 2308-2312. https://doi.org/10.1111/jgs.12000

Katsumi, Y., Andreano, J. M., Barrett, L. F., Dickerson, B. C., & Touroutoglou, A. (2021). Greater Neural Differentiation in the Ventral Visual Cortex Is Associated with Youthful Memory in Superaging. *Cerebral Cortex*, *31*(11), 5275-5287. https://doi.org/10.1093/cercor/bhab157

Katsumi, Y., Wong, B., Cavallari, M., Fong, T. G., Alsop, D. C., Andreano, J. M., Carvalho, N., Brickhouse, M., Jones, R., Libermann, T. A., Marcantonio, E. R., Schmitt, E., Shafi, M. M., Pascual-Leone, A., Travison, T., Barrett, L. F., Inouye, S. K., Dickerson, B. C., & Touroutoglou, A. (2022). Structural integrity of the anterior mid-cingulate cortex contributes to resilience to delirium in SuperAging. *Brain Communications*, *4*(4). https://doi.org/10.1093/braincomms/fcac163

Keenan, H. E., Czippel, A., Heydari, S., Gawryluk, J. R., & Mazerolle, E. L. (2024). Intrinsic functional connectivity strength of SuperAgers in the default mode and salience networks: Insights from ADNI. *Aging Brain*, *5*. https://doi.org/10.1016/j.nbas.2024.100114

Kim, B. R., Kwon, H., Chun, M. Y., Park, K. D., Lim, S. M., Jeong, J. H., & Kim, G. H. (2020). White Matter Integrity Is Associated With the Amount of Physical Activity in Older Adults With Super-aging. *Frontiers in Aging Neuroscience*, *12*. https://doi.org/10.3389/fnagi.2020.549983

Kim, H. E., Kim, B. R., Hong, S. H., Song, S. Y., Jeong, J. H., & Kim, G. H. (2024). Predicting superagers: a machine learning approach utilizing gut microbiome features. *Frontiers in Aging Neuroscience*,

16. https://doi.org/10.3389/fnagi.2024.1444998

Klinedinst, B. S., Kharate, M. K., Mohammadiarvejeh, P., Fili, M., Pollpeter, A., Larsen, B. A., Moody, S., Wang, Q., Allenspach, K., Mochel, J. P., & Willette, A. A. (2023). Exploring the secrets of super-aging: a UK Biobank study on brain health and cognitive function. *GeroScience*, *45*(4), 2471-2480. https://doi.org/10.1007/s11357-023-00765-x

Lin, F. V., Ren, P., Mapstone, M., Meyers, S. P., Porsteinsson, A., & Baran, T. M. (2017). The cingulate cortex of older adults with excellent memory capacity. *Cortex*, *86*, 83-92. https://doi.org/10.1016/j.cortex.2016.11.009

Lin, F. V., Wang, X., Wu, R., Rebok, G. W., & Chapman, B. P. (2017). Identification of Successful Cognitive Aging in the Alzheimer's Disease Neuroimaging Initiative Study. *Journal of Alzheimer's Disease*, *59*(1), 101-111. https://doi.org/10.3233/jad-161278

Linuma, Y., Nobukawa, S., Mizukami, K., Kawaguchi, M., Higashima, M., Tanaka, Y., Yamanishi, T., & Takahashi, T. (2022). Enhanced temporal complexity of EEG signals in older individuals with high cognitive functions. *Frontiers in Neuroscience*, *16*. https://doi.org/10.3389/fnins.2022.878495

Mapstone, M., Lin, F., Nalls, M. A., Cheema, A. K., Singleton, A. B., Fiandaca, M. S., & Federoff, H. J. (2017). What success can teach us about failure: the plasma metabolome of older adults with superior memory and lessons for Alzheimer's disease. *Neurobiology of Aging*, *51*, 148-155. https://doi.org/10.1016/j.neurobiolaging.2016.11.007

Nassif, C., Kawles, A., Ayala, I., Minogue, G., Gill, N. P., Shepard, R. A., Zouridakis, A., Keszycki, R., Zhang, H., Mao, Q., Flanagan, M. E., Bigio, E. H., Mesulam, M. M., Rogalski, E., Geula, C., & Gefen, T. (2022). Integrity of Neuronal Size in the Entorhinal Cortex Is a Biological Substrate of Exceptional Cognitive Aging. *The Journal of Neuroscience*, *42*(45), 8587-8594. https://doi.org/10.1523/jneurosci.0679-22.2022

Park, C.-h., Kim, B. R., Park, H. K., Lim, S. M., Kim, E., Jeong, J. H., & Kim, G. H. (2022). Predicting superagers by machine learning classification based on the functional brain connectome using resting-state functional magnetic resonance imaging. *Cerebral Cortex*, *32*(19), 4183-4190. https://doi.org/10.1093/cercor/bhab474

Park, J., Lee, Y., & Won, C. W. (2022). CEND1 and miR885 methylation changes associated with successful

cognitive aging in community-dwelling older adults. *Experimental Gerontology*, *160*. https://doi.org/10.1016/j.exger.2022.111704

Park, J., Won, C. W., Saligan, L. N., Kim, Y.-J., Kim, Y., & Lukkahatai, N. (2021). Accelerated Epigenetic Age in Normal Cognitive Aging of Korean Community-Dwelling Older Adults. *Biological Research For Nursing*, *23*(3), 464-470. https://doi.org/10.1177/1099800420983896

Patel, R., Cosentino, S., Zheng, E. Z., Schupf, N., Barral, S., Feitosa, M., Andersen, S. L., Sebastiani, P., Ukraintseva, S., Christensen, K., Zmuda, J., Thyagarajan, B., & Gu, Y. (2024). Systemic inflammation in relation to exceptional memory in the Long Life Family Study (LLFS). *Brain, Behavior, & Immunity - Health*, *37*. https://doi.org/10.1016/j.bbih.2024.100746

Pezzoli, S., Giorgio, J., Martersteck, A., Dobyns, L., Harrison, T. M., & Jagust, W. J. (2023). Successful cognitive aging is associated with thicker anterior cingulate cortex and lower tau deposition compared to typical aging. *Alzheimer's & Dementia*, *20*(1), 341-355. https://doi.org/10.1002/alz.13438

Pudas, S., Persson, J., Josefsson, M., de Luna, X., Nilsson, L.-G., & Nyberg, L. (2013). Brain Characteristics of Individuals Resisting Age-Related Cognitive Decline over Two Decades. *The Journal of Neuroscience*, *33*(20), 8668-8677. https://doi.org/10.1523/jneurosci.2900-12.2013

Riis, J. L., Chong, H., Ryan, K. K., Wolk, D. A., Rentz, D. M., Holcomb, P. J., & Daffner, K. R. (2008). Compensatory neural activity distinguishes different patterns of normal cognitive aging. *NeuroImage*, *39*(1), 441-454. https://doi.org/10.1016/j.neuroimage.2007.08.034

Rosano, C., Aizenstein, H. J., Newman, A. B., Venkatraman, V., Harris, T., Ding, J., Satterfield, S., & Yaffe, K. (2012). Neuroimaging differences between older adults with maintained versus declining cognition over a 10-year period. *NeuroImage*, *62*(1), 307-313. https://doi.org/10.1016/j.neuroimage.2012.04.033

Silverman, J. M., Schmeidler, J., Beeri, M. S., Rosendorff, C., Sano, M., Grossman, H. T., Carrión-Baralt, J. R., Bespalova, I. N., West, R., & Haroutunian, V. (2012). C-reactive protein and familial risk for dementia A phenotype for successful cognitive aging. *Neurology*, *79*(11), 1116-1123. https://doi.org/DOI 10.1212/WNL.0b013e3182698c89

Spencer, B. E., Banks, S. J., Dale, A. M., Brewer, J. B., Makowski-Woidan, B., Weintraub, S., Mesulam, M.

M., Geula, C., & Rogalski, E. (2022). Alzheimer's polygenic hazard score in SuperAgers: SuperGenes or SuperResilience? *Alzheimer's & Dementia: Translational Research & Clinical Interventions*, *8*(1). https://doi.org/10.1002/trc2.12321

Sun, F. W., Stepanovic, M. R., Andreano, J., Barrett, L. F., Touroutoglou, A., & Dickerson, B. C. (2016). Youthful Brains in Older Adults: Preserved Neuroanatomy in the Default Mode and Salience Networks Contributes to Youthful Memory in Superaging. *The Journal of Neuroscience*, *36*(37), 9659-9668. https://doi.org/10.1523/jneurosci.1492-16.2016

Tobe, M., Nobukawa, S., Mizukami, K., Kawaguchi, M., Higashima, M., Tanaka, Y., Yamanishi, T., & Takahashi, T. (2023). Hub structure in functional network of EEG signals supporting high cognitive functions in older individuals. *Frontiers in Aging Neuroscience*, *15*. https://doi.org/10.3389/fnagi.2023.1130428

Waiter, G. D., Fox, H. C., Murray, A. D., Starr, J. M., Staff, R. T., Bourne, V. J., Whalley, L. J., & Deary, I. J. (2008). Is retaining the youthful functional anatomy underlying speed of information processing a signature of successful cognitive ageing? An event-related fMRI study of inspection time performance. *NeuroImage*, *41*(2), 581-595. https://doi.org/10.1016/j.neuroimage.2008.02.045

Wang, L., & Zhang, Z. (2021). Classification of longitudinal brain networks with an application to understanding superior aging. *Stat*, *10*(1). https://doi.org/10.1002/sta4.402

Wang, X., Ren, P., Baran, T. M., Raizada, R. D. S., Mapstone, M., & Lin, F. (2019). Longitudinal Functional Brain Mapping in Supernormals. *Cerebral Cortex*, *29*(1), 242-252. https://doi.org/10.1093/cercor/bhx322

Xu, X., Lin, L., Wu, S., & Sun, S. (2023). Exploring Successful Cognitive Aging: Insights Regarding Brain Structure, Function, and Demographics. *Brain Sciences*, *13*(12). https://doi.org/10.3390/brainsci13121651

Yang, Y., Chen, Y., Sang, F., Zhao, S., Wang, J., Li, X., Chen, C., Chen, K., & Zhang, Z. (2022). Successful or pathological cognitive aging? Converging into a “frontal preservation, temporal impairment (FPTI)” hypothesis. *Science Bulletin*, *67*(22), 2285-2290. https://doi.org/10.1016/j.scib.2022.11.004

Zhang, J., Andreano, J. M., Dickerson, B. C., Touroutoglou, A., & Barrett, L. F. (2020). Stronger Functional

Connectivity in the Default Mode and Salience Networks Is Associated With Youthful Memory in Superaging. *Cerebral Cortex*, *30*(1), 72-84. https://doi.org/10.1093/cercor/bhz071
